# Chlorophyll-Derivative Modulation of Rhodopsin Signaling Properties through Evolutionarily Conserved Interaction Pathways

**DOI:** 10.3389/fmolb.2017.00085

**Published:** 2017-12-12

**Authors:** Kristina N. Woods, Jürgen Pfeffer, Judith Klein-Seetharaman

**Affiliations:** ^1^Lehrstuhl für BioMolekulare Optik, Ludwig-Maximilians-Universität, München, Germany; ^2^Bavarian School of Public Policy, Technical University of Munich, München, Germany; ^3^Warwick Medical School, University of Warwick, Coventry, United Kingdom

**Keywords:** Terahertz spectroscopy, protein allostery, small-molecule allosteric agonist, Chlorine Compounds, protein dynamics

## Abstract

Retinal is the light-absorbing chromophore that is responsible for the activation of visual pigments and light-driven ion pumps. Evolutionary changes in the intermolecular interactions of the retinal with specific amino acids allow for adaptation of the spectral characteristics, referred to as spectral tuning. However, it has been proposed that a specific species of dragon fish has bypassed the adaptive evolutionary process of spectral tuning and replaced it with a single evolutionary event: photosensitization of rhodopsin by chlorophyll derivatives. Here, by using a combination of experimental measurements and computational modeling to probe retinal-receptor interactions in rhodopsin, we show how the binding of the chlorophyll derivative, chlorin-e6 (Ce6) in the intracellular domain (ICD) of the receptor allosterically excites G-protein coupled receptor class A (GPCR-A) conserved long-range correlated fluctuations that connect distant parts of the receptor. These long-range correlated motions are associated with regulating the dynamics and intermolecular interactions of specific amino acids in the retinal ligand-binding pocket that have been associated with shifts in the absorbance peak maximum (λ_max_) and hence, spectral sensitivity of the visual system. Moreover, the binding of Ce6 affects the overall global properties of the receptor. Specifically, we find that Ce6-induced dynamics alter the thermal stability of rhodopsin by adjusting hydrogen-bonding interactions near the receptor active-site that consequently also influences the intrinsic conformational equilibrium of the receptor. Due to the conservation of the ICD residues amongst different receptors in this class and the fact that all GPCR-A receptors share a common mechanism of activation, it is possible that the allosteric associations excited in rhodopsin with Ce6 binding are a common feature in all class A GPCRs.

## Introduction

The dimmest habitats on earth appear at night and in the depths of the ocean (Warrant, 2004). The greatest challenge for vision in these habitats is capture of photons, and the way these photons are post-processed. The primary photoreceptor in eyes, rhodopsin, is a major target for adaptation to different light conditions. Rhodopsin as the prototypical member of the GPCR-A family adopts an overall organization of seven transmembrane (TM) helices that form a bundle. Within the GPCR family there is a large sub-group of opsins, representing opsin sequences in different photoreceptor cell types and organisms. All opsins covalently bind retinal, a vitamin A derivative, at the interface between the transmembrane (TM) and extracellular domains (ECDs). Visual signal transduction is initiated by photon-induced isomerization of *11-cis* retinal to *all-trans* retinal. This event is sensed by the TM domain which undergoes a conformational change that results in the activated state, Metarhodopsin II (Meta-II) via Meta-I. Meta-II—unlike dark state rhodopsin—binds and activates the G protein, transducin G_t_, ultimately leading to receptor hyperpolarization. Signal desensitization is initiated by phosphorylation of the C-terminus of rhodopsin by rhodopsin kinase, followed by binding of arrestin, preventing further binding of G_t_ to rhodopsin. *In vitro*, Meta-II decays to opsin and free retinal, the half-life of which depends on the lipid environment (Farrens and Khorana, 1995). In addition, a storage form of rhodopsin, Meta-III, emerges in parallel with Meta-II, from the Meta-I state. The Meta-III state of rhodopsin—unlike Meta-II—has a protonated retinal Schiff base and decays into opsin and free retinal on significantly longer time scales (Heck et al., 2003b,a; Vogel et al., 2003, 2004; Stehle et al., 2014).

One mechanism of adaptation to dim habitats is spectral tuning. Spectral tuning refers to adjustment of the absorbance maxima in the spectra of the photoreceptors. Spectral tuning can be hard-wired by genetic variation of the rhodopsin sequences changing the interactions between the retinal and the protein (“opsin shift”) (Nathans, 1990). This allows adaptation to the wavelengths that are maximally transmitted under different environmental conditions, such as sun-light in the shade, sun-light penetrating water, moon-light or bioluminescence generated by deep-sea fish (Douglas et al., 1998; Fishkin et al., 2004). Genetic adaptation is also the mechanism by which cone and rod opsins absorb at different wavelengths (Douglas et al., 1999). However, spectral tuning can also be achieved by interaction of the rhodopsins with small molecules. In xanthorhodopsin, a bacteriorhodopsin-like proton pump in the halophilic eubacterium *Salinibacter ruber*, a carotenoid is bound in addition to retinal (Balashov et al., 2005). Light energy absorbed by the carotenoid is transferred to the retinal with a quantum efficiency of ~40% (Balashov et al., 2005) and light is funneled to the retinal similar to the photosynthetic light harvesting complex. In the deep-sea fish *Malacosteus niger* (dragon fish), porphyrin/chlorophyll derivatives, including chlorin e6 (Ce6), may act as photosensitizers rendering its rhodopsin sensitive to wavelengths that other deep-sea fish respond to as a result of genetic adaptations of their retinal binding pockets or retinal replacement (Douglas et al., 1998, 1999; Isayama et al., 2006). An investigation of different chlorophyll derivatives in rod outer segments on bleaching rates of the chromophore by red light highlighted Ce6 as the molecule with the strongest photosensitizing effect on bovine rhodopsin (Washington et al., 2004). The hypothesis of a binding pocket for Ce6 and energy transfer to retinal resembling that of light harvesting in photosynthesis was proposed (Washington et al., 2004; Isayama et al., 2006). The photosensitizing effects of chlorophyll-derivatives observed in salamander and deep-sea fish can be reproduced qualitatively using bovine rhodopsin, *in vitro* (Washington et al., 2004). Furthermore, electroretinogram recordings in mice injected with Ce6 suggested that the sensitivity of rhodopsin can be broadened to blue and red light in the presence of Ce6 (Washington et al., 2007). Indeed, the photosensitizing effect of Ce6 and other porphyrin compounds in vision has been reported as a side-effect during photodynamic therapy (Kimura, 1987).

To understand by what Ce6 binding enhances bleaching rates of rhodopsin in red light, we used a novel approach which we recently developed to probe dynamics and allostery in rhodopsin (Woods et al., 2016): through a combination of molecular dynamics simulations, evolutionary sequence analysis and Terahertz (THz) spectroscopy we show that Ce6 binding excites evolutionarily conserved communication pathways in rhodopsin that establish connections between the ligand-binding site and the rest of the receptor.

## Results

### Experimental detection of the conformational ensemble dynamics and intermolecular changes in rhodopsin when bound with the allosteric modulator Ce6

#### Global fluctuations of dark-state rhodopsin bound with Ce6

**Figure 1:**
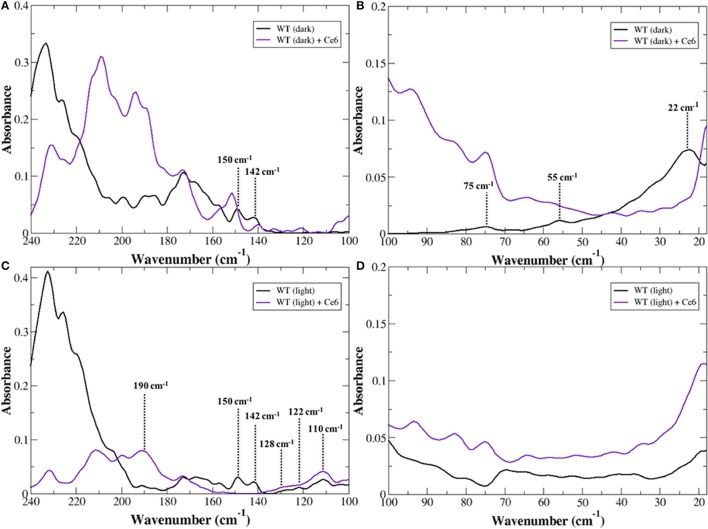
Experimental THz spectrum of dark-state rhodopsin in the unbound state (black line) and when bound with Ce6 (purple line) in the **(A)** 240–100 cm^−1^ spectral region and in the **(B)** 100–20 cm^−1^, spectral region. Experimental THz spectrum of Meta-II in the unbound state (black line) and when bound with Ce6 (purple line) in the **(C)** 240–100 cm^−1^ spectral region and in the **(D)** 100–20 cm^−1^ spectral region.

The <100 cm^−1^ region of the infrared spectrum is sensitive to global, internal fluctuations that describe the intrinsic dynamics of the receptor. These globally, correlated thermal fluctuations provide a mechanism for sampling the ensemble of conformations that comprise the free energy landscape of possible receptor conformations (Frauenfelder et al., 1991). Hence, the modes detected in the experimental THz spectrum in this region provide direct information about the sampling of conformational substates in rhodopsin. An inspection of the low frequency modes of dark-state rhodopsin in the <100 cm^−1^ spectral region in Figure 1A in the presence and absence of Ce6 reveals that the region of the spectrum above 50 cm^−1^ is dramatically altered when contrasting the two states of the receptors. For instance, in the Ce6-bound receptor in Figure 1B there is a prominent peak at 80 cm^−1^ and a general increase in the absorption peak intensity at a frequency above 50 cm^−1^ when contrasted with the dark-state receptor that is not bound with Ce6 (Figure 1 and Woods et al., 2016). The differences in the spectra of the two receptors suggest that binding of Ce6 alters the conformational ensemble dynamics in rhodopsin.

In our previous work on the inactive-receptor in the unbound state (Woods et al., 2016), we have found there is an equilibrium of both inactive and active-state protein conformational fluctuations in the dark-state protein. This conformational heterogeneity in the inactive receptor indicates that rhodopsin samples a diverse set of functional structures even before any activation event has taken place. In particular, the heterogeneity of global structural fluctuations detected experimentally is intimately tied with multiple *pre-existing* allosteric associations in the inactive receptor. Peaks at approximately 75 and 55 cm^−1^ in the experimental spectrum of the unbound receptor were identified as rhodopsin global fluctuations in an inactive-type conformation. Specifically, the peak at 55 cm^−1^ was found to be associated with a retinal (polyene chain) torsional fluctuation that is coupled with a protein, global backbone torsion, whereas the peak at 75 cm^−1^ is associated with a retinal torsional oscillation that is coupled with collective out-of-plane protein side-chain fluctuations (Woods et al., 2016). In addition to the inactive-state conformational fluctuations, we also uncovered a weaker band at approximately 40 cm^−1^ in the experimental spectrum that was later found to be associated with transient interactions of receptor amino acid side-chains that supported a more active-like rhodopsin conformation. These retinal-induced transient interactions of the more active-like receptor conformation were hypothesized to serve as a necessary precursor in the mechanism that eventually leads to the active-state receptor. Therefore, the enhancement of the ~75 cm^−1^ mode in the Ce6-bound receptor suggests that Ce6 stabilizes a specific conformational state of rhodopsin—the ground state.

#### Ce6-induced intra- and inter-protein interaction networks in dark-state rhodopsin

In the 100–250 cm^−1^ region of the experimental spectrum we detect motions in rhodopsin that reveal more localized intermolecular interactions (Woods, 2014b) such as interhelical contacts as well as helical interactions with the solvent. In general, peaks in the experimental spectrum in the 100–160 cm^−1^ are related to protein intra- and intermolecular interactions. For example in Figure 1A, the peaks at approximately 150 and 140 cm^−1^ in the dark-state spectrum of the unbound receptor were found to be associated with interhelical and solvent-induced H-bonding interactions in our earlier study (Woods et al., 2016), while peaks ≥170 cm^−1^ are predominately associated with solvent-solvent interactions of water molecules in the receptor hydration shell. A comparison of dark-state rhodopsin in the unbound and unbound state in Figure 1A suggests that binding of Ce6 alters the inter- and intra-protein interactions in rhodopsin but has a much stronger effect on an extensive network of solvent H-bonds that stabilize the ground state of the receptor (Woods, 2014a).

### MD simulation of Ce6-induced long-range correlated fluctuations and the effect on dark-state rhodopsin global motions

**Figure 2:**
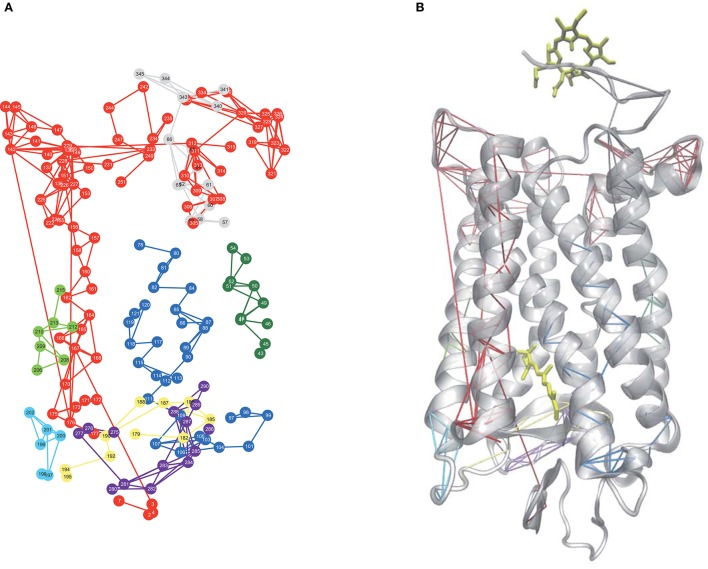
**(A)** A 2-D network mapping of the LSFs from the MD simulation of dark-state rhodopsin when bound to Ce6 and **(B)** a cartoon representation of rhodopsin showing the mapping of the LSFs from **(A)** onto the protein 3-D structure.

**Figure 3:**
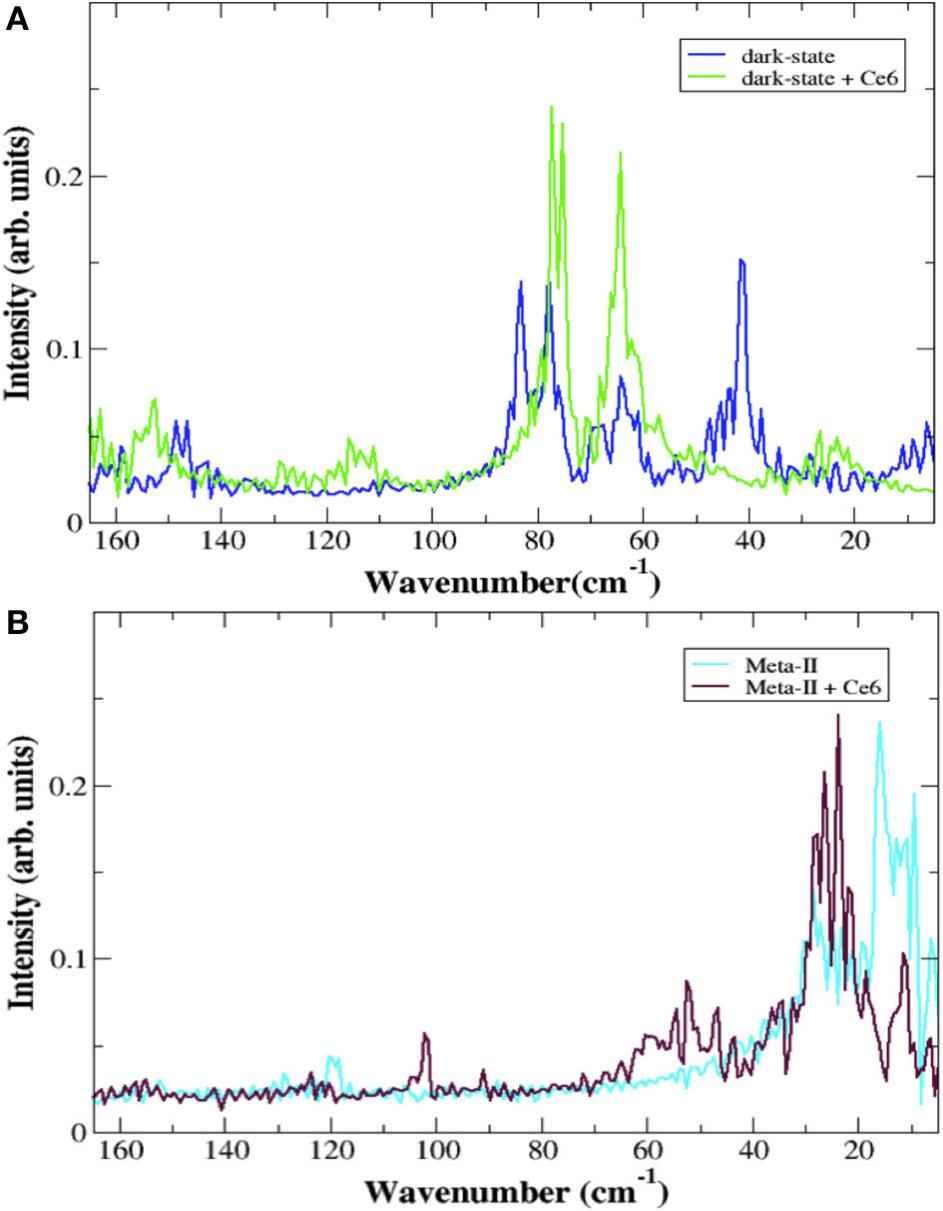
The torsional spectrum of the retinal from rhodopsin MD simulations in **(A)** the dark-state (blue line) and in the dark-state when bound to Ce6 (green line) and **(B)** in Meta-II (cyan line) and Meta-II when bound to Ce6 (purple line). The peaks at 15 and 25 cm^−1^ in **(B)** are related to torsional fluctuations of the C-20 methyl group near the terminus of the polyene chain and a collective chain-twisting oscillation, respectively in Meta-II whereas the peak close to 60 cm^−1^ in **(B)** is associated with a chain torsion coupled with a retinal ring bending motion of a more inactive-type rhodopsin structure. In all cases, the torsion of the retinal is defined by the angle created by the C5-, C9-, and C13- methyl groups.

Allostery in proteins enables the activity of one site in a protein to modulate function at another spatially distinct region (Hawkins and McLeish, 2006; Fenwick et al., 2011; Motlagh et al., 2014). Recent experimental and computation investigations on a number of proteins and enzymes have demonstrated that allosteric signal transmission is mediated by protein local structural fluctuations (Whitten et al., 2005; Daily and Gray, 2007; Pandini et al., 2013). To assess if Ce6 allosterically affects retinal-protein interactions in rhodopsin, we carried out MD simulations of dark-state bound rhodopsin in the presence and absence of Ce6. We find that Ce6 binds only weakly on the cytoplasmic surface (Supplementary text, *Ce6–ligand binding affinity* and Figures S2, S3) of the receptor. Experimental fluorescence measurements of Ce6 binding to rhodopsin also reveal micromolar binding affinity (unpublished results). Despite the weak binding, Ce6 has a strong effect on the long-range correlated fluctuations of the bound state when contrasted with the unbound receptor. This can be deduced from our analysis of the MD simulation induced localized structural fluctuations (LSFs) and the consequent collective dynamics in the receptor that arise from the addition of Ce6 to rhodopsin. For instance, Figure 2 reveals the development of long-range correlated fluctuations in dark-state rhodopsin with Ce6 that significantly involve contributions from the intracellular loops close to where Ce6 binding takes place. Particularly, Ce6-induced long-range correlated fluctuations involving intracellular loop 2 (CL2) and extracellular loop 2 (EL2) are coupled with a structurally conserved collection of aromatic and polar residues at the extracellular end of H4 (Pro170, Pro171, Tyr175, Ser176, Arg177, and Tyr178) leading from the ligand-binding pocket to the EC domain. This group of GPCR-A wide conserved long-distance fluctuations modulates the H-bonding environment of residues and solvent molecules lining the retinal ligand-binding pocket (Figure 2B). Specifically, side-chain fluctuations of residues such as Glu122, Trp126, and Phe261 in Ce6 rhodopsin have increased (H-bonding) interactions with the retinal β-ionone ring due to the CL2–EL2 correlation when contrasted with unbound receptor (Figure S1b). The Ce6-induced correlated fluctuations reorient Glu122 so that the there is a rearrangement of the H-bonding network surrounding the ring such that it stabilizes the retinal in a defined conformation (Figure 2 and Figure S4). In our previous work (Woods et al., 2016) we have conjectured that the ability of the retinal to assume multiple conformational states is intertwined with the formation of multiple, distinct signaling pathways in both the inactive and active receptor. In line with what was observed in the experimental detection of the global modes of the dark-state receptor in Figure 1, we find from the MD analysis of the Ce6-induced structural fluctuations in rhodopsin, that binding of the allosteric modulator assists in stabilizing the receptor in a specific conformation, namely the ground state because of the modified electrostatic interactions in the vicinity of the retinal ring that accompany Ce6-binding. This conclusion is further supported by a mapping of the low-frequency torsional dynamics of the retinal from the dark-state MD simulations of rhodopsin in both the unbound and bound states. The absence of the 40 cm^−1^ mode in the MD retinal torsional spectrum of Ce6-bound rhodopsin in Figure 3A clearly shows that Ce6 stabilizes only a subset of the receptor conformations. Both the 80 and 65 cm^−1^ represent the effect of retinal torsional dynamics from interactions with the inactive-like conformation of rhodopsin, whereas the 40 cm^−1^ mode reflects the influence on the retinal from protein interactions in a more active-like conformation (Woods et al., 2016).

Ce6-induced long-distance induced fluctuations involving CL1 and CL3 are instrumental in altering the interhelical packing within the receptor core. Correlated fluctuations involving these two loops modulate the dynamics of TM1and TM2 (Figure 2) and subsequently destabilize a conserved cluster of water molecules near Gly90 in helix 2 that maintain the shape of the ligand-binding pocket. In particular, the Ce6-induced distortion of the binding pocket weakens interactions in the ligand-binding region that stabilize residues forming the receptor hydrophobic core such as Gly114, Ala117, Thr118, and Gly120–Glu122 and concurrently, also disrupts a conserved network (Angel et al., 2009) of water molecules near Gly90 in helix 2 that stabilizes the Glu113 salt bridge of the protonated Schiff base (PSB). These Ce6-induced changes within the receptor ligand-binding pocket allow Glu113 to move further away from the PSB. This perturbation of the PSB H-bonding network increases the amplitude of the dynamic structural fluctuations of the counterion and affects the thermal stability of the entire receptor. The reduced electrostatic interactions from residues surrounding the PSB, resulting from the movement of Glu113 away from the PSB, decreases the energy gap between the ground and excited state of the receptor (Rajamani et al., 2011). The reduction of the energy difference between the two states also reduces the barrier for activation; hence, it reduces receptor thermal stability (Figure S4). Numerous previous studies on rhodopsin (Lin et al., 1998; Rajamani et al., 2011; Imamoto and Shichida, 2014) have also shown that the reduction in the energy difference between the ground and excited state reduces the thermal stability of the receptor and is also directly correlated with the red-shift of the λ_max_ of the receptor. Although, it is important to point out that previous thermal denaturing studies (Balem et al., 2009) on inactive-state rhodopsin found an increase in the helical content of the receptor when bound to Ce6. In other words, the helical regions of the receptor are stabilized in the dark-state receptor when bound to Ce6 (when contrasted with the unbound receptor). These results are in direct contrast to both the MD simulation studies and (THz) experimental studies carried out in this investigation. Although, the disparity in interpretations could be a consequence of what the distinctive experimental methods measure. The melting data is primarily sensitive to changes in the helical content of the receptor, whereas the THz data (≤100 cm^−1^) is sensitive to the global changes in the receptor as a whole. The initial findings from this investigation suggest that the principal changes in thermal stability arise from modifications around the ligand-binding site in the Ce6-bound receptor and these alterations influence the global stability of the receptor.

The deformation of the retinal ligand-binding pocket due to the Ce6-induced correlated fluctuations of CL1 and CL3 is further stabilized by the rearrangement of conserved network of water molecules in EL2. An analysis of the CL1-CL3 Ce6-induced correlated dynamics in Figure 2 also reveals alterations in solvent-protein interactions that include the water molecules shared between the side-chains of Glu181 and Ser186 that directly connect the dynamics of the EL2 with the retinal binding-pocket (Figure S5). The CL1-CL3 coordinated dynamics shift Glu181 further away from Ser186 by means of the introduction of an additional water molecule into the retinal binding pocket that also links the backbone atoms of Glu181 and Ser186 (while retaining the water coordinated side-chain linkage of the two residues). This results in the overall rearrangement of EL2 through the correlated dynamics of helix 3 via the conserved Cys110-Cys187 disulfide bond. The correlated movement shifts Ile189 (as well as the entire β_4_ loop of EL2) in the direction of the receptor N-terminus. The end result of the altered Ce6-induced dynamics of dark-state rhodopsin is a more open ligand-binding pocket that is created by the larger separation between the retinal and extracellular region of the receptor. The increased distance between Ile189 and the retinal disrupts the H-bonding network of the PSB but in doing so, would also likely lead to a decrease in receptor thermal stability (associated with higher levels of dark-noise) as well as increase the rate of hydrolysis of the PSB. In fact, earlier experimental studies (Imamoto and Shichida, 2014; Yanagawa et al., 2015) focusing on the thermal activation rate of rhodopsin have revealed a direct correlation between the thermal stability of the dark-state of the receptor and that of the lifetime of the active-state intermediate Meta-II. In this regard, both Glu122 and Ile189 were identified as two residues that play a crucial role in suppressing thermal fluctuations in the retinal-binding pocket in rhodopsin, which ultimately imparts the low dark noise characteristic of the receptor.

It is also interesting to return to the experimental detection of the global modes of the dark-state receptor (Figure 1A) and to consider the observed changes in the conformational ensemble dynamics that were detected with Ce6-binding. Previous experimental studies probing the conformational stability of Ce6 in (dark-state) rhodopsin have indicated that Ce6 stabilizes the helices of the receptor. This earlier work is in line with the experimentally detected population shift in the conformational ensemble dynamics of the Ce6-bound receptor (when contrasted with the unbound receptor) that we have detected in this investigation on rhodopsin (Figure 1A). But interestingly, is in direct contrast to the identified changes in the ligand-binding pocket dynamics of the bound receptor that we have uncovered from MD simulation. In the latter case, the Ce6-induced dynamics appears to create a closer potential energy surface between the ground and excited state (Ala-Laurila et al., 2004; Hofmann and Palczewski, 2015) of the retinal in Ce6-rhodopsin. This reduction in the energy difference between the two states would, in principal, create a thermally unstable ground state that consequently would shift the equilibrium toward the excited-state of the receptor. The disparity in interpretations that arise when examining the local dynamics of the retinal-binding pocket with approaches that map the global characteristics of the receptor suggests that induced long-range structural fluctuations may also play an important role in the Ce6 mechanism of spectral tuning in rhodopsin.

### Experimental detection of the induced changes in global dynamics and the allosterically coupled motions associated with activation in Meta-II rhodopsin when bound by Ce6

#### Global dynamics and thermal stability in Meta-II-Ce6

The global motions of Meta-II rhodopsin in the presence of Ce6 are dramatically altered as compared to dark rhodopsin with and without Ce6 in Figure 1D. In contrast to the dark-state unbound spectrum in Figure 1B, there are no clearly resolved vibrational bands in the <100 cm^−1^ region of the experimental spectrum. Based on previous work on rhodopsin (Woods et al., 2016), this likely indicates that the major protein modes excited after isomerization are red-shifted to very low frequencies. Despite the lack of spectral features, one can still deduce information about the stability of the receptor states based on the general shape of the spectrum. For instance, it is apparent that the Ce6-bound Meta-II state in Figure 1C is far more oscillatory above 70 cm^−1^ when contrasted with the unbound Meta-II state. The oscillatory nature of the Meta-II-Ce6 spectrum indicates that there is instability in the internal modes of the receptor. Moreover, the increased instability detected in the global modes of the Ce6-bound receptor also implies a general decrease in the thermal stability of the Ce6-bound activated-state of the receptor.

#### Experimental detection of alterations in the allosteric interaction network associated with activation in Meta-II-Ce6

An inspection of the higher frequency spectra of the Meta-II states of rhodopsin in Figure 1C strongly suggests that there are major modifications in the interhelical interactions in Meta-II-Ce6 when compared with the unbound active-state receptor. For example, the absence of the ~140 and 150 cm^−1^ modes in the Ce6 receptor bound spectrum suggests that both the interhelical packing and the solvent-protein interactions in the bound receptor are dramatically altered in Meta-II-Ce6. Both modes (at 140 and 150 cm^−1^) are strongly influenced by retinal torsional dynamics associated with both the β-ionone ring and the polyene chain, indicating that the coupling between the retinal ligand and its immediate protein environment is considerably changed in the Ce6-activated state. In our earlier study (Woods et al., 2016) of Meta-II rhodopsin we determined that the vibrational modes at approximately 110, 120, and 130 cm^−1^ are spectral markers of the water-mediated pathway of activation in Meta-II that connects the dynamics in the ligand-binding pocket with the G-protein binding site in the ICD. The vibrational modes represent anharmonic, solvent-mediated fluctuations of protein backbone and side-chain atoms that form allosteric sites in Meta-II. The water-mediated allosteric sites create a coherent, defined pathway of signal propagation in Meta-II that connect the dynamics of the retinal taking place in the ligand-binding pocket with intermolecular interactions taking place in the receptor intracellular domain. The fact that both spectra in Figure 1C have prominent vibrational bands at about 110, 120, and 130 cm^−1^ implies that activation in the Ce6 bound receptor still takes place, but based on the other prominent differences in the spectrum of the distinct Meta-II states, it is likely that the activation mechanism in Meta-II-Ce6 is somehow altered. One clue of how the pathway of activation in Meta-II-Ce6 may differ from Meta-II can be discerned from the solvent—solvent interaction region of the experimental spectrum (>170 cm^−1^) in Figure 1C. A comparison of the spectra makes it readily apparent that solvent interaction network in Meta-II is disrupted with Ce6 binding. The dramatic drop in intensity in Meta-II-Ce6 in the 200–240 cm^−1^ spectral region suggests that the addition of the allosteric modulator (Ce6) disrupts the solvent H-bonding network that both stabilizes and aids in the efficiency of the active Meta-II intermediate binding with the G-protein. Although interestingly, in the same solvent-interaction region, there is a new peak in the Meta-II-Ce6 spectrum centered at 190 cm^−1^ that possibly hints at a new set of solvent interactions that support an alternative pathway of activation in the Ce6 bound receptor. In previous THz studies on globular proteins (Woods, 2010, 2014a) we have identified an equivalent peak at approximately 190 cm^−1^ in the protein-solvent coupling region of the experimental spectrum that describes interaction dynamics directly tied with the formation of long-range communication channels in the protein. Particularly, in the previous instances we found that the anharmonic dynamics of the solvent molecules within the hydration shell coupled with protein motions to promote long-range coherence pathways in the protein three-dimensional structure.

### MD simulation of Meta-II bound with Ce6

#### Localized structural fluctuations in Meta-II-Ce6 and the disruption of the active-state activation pathway

**Figure 4:**
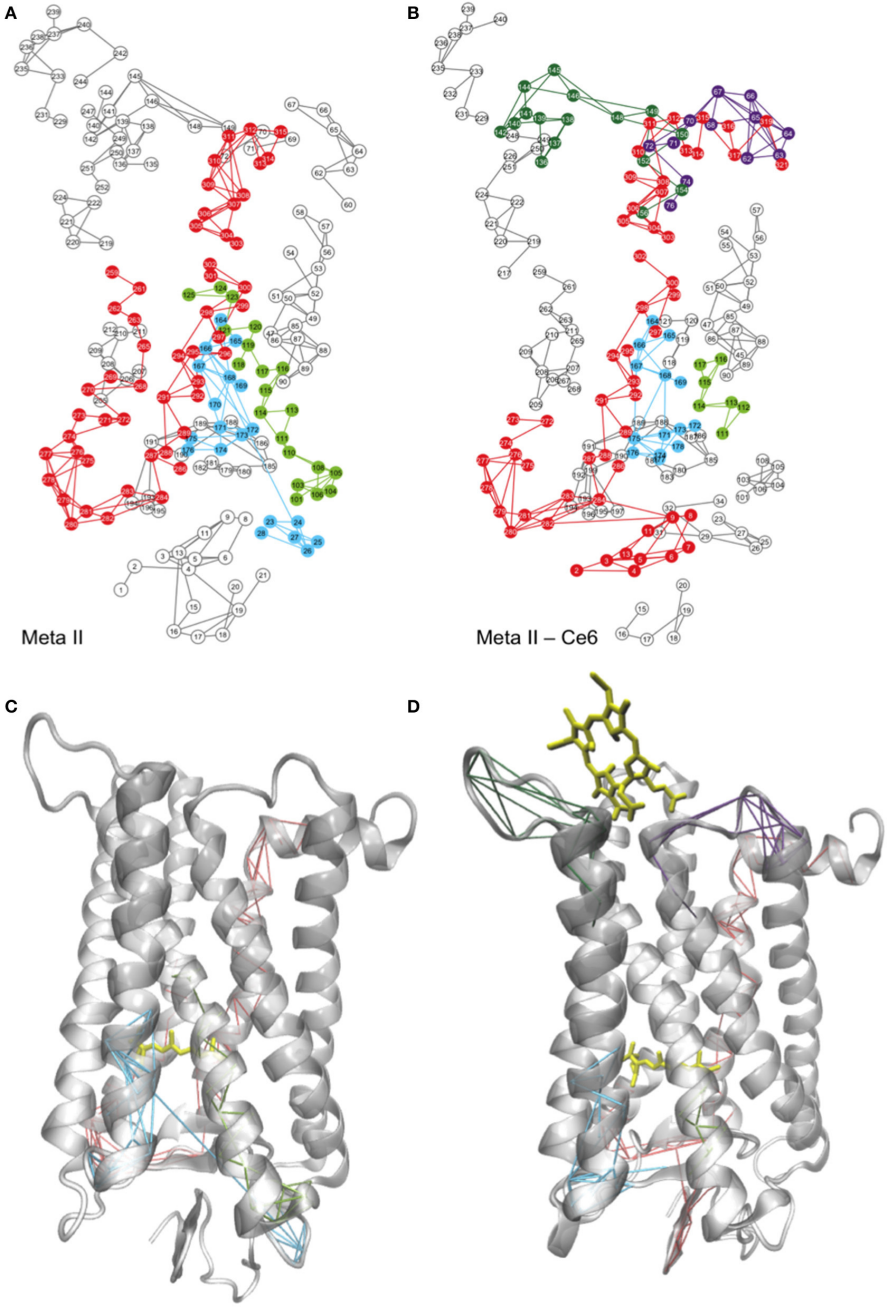
A 2-D mapping of the LSFs of **(A)** Meta-II and **(B)** Meta-II-Ce6 from the MD simulations of rhodopsin and the corresponding mapping of the LSFs onto a cartoon representation of the 3-D structure of **(C)** Meta-II and **(D)** Meta-II-Ce6.

A network representation of the Meta-II LSFs from the MD simulations of Ce6-bound and -unbound rhodopsin structures are shown in Figures 4A,B and a mapping of the LSF components onto the two Meta-II structures is shown in Figures 4C,D. One of the distinguishing features that we detect in the Meta-II-Ce6 LSF when contrasted with unbound Meta-II is a disruption in the structural overlap of interactions that creates an activation pathway from the ligand-binding region to the cytoplasmic surface. Specifically, in the Meta-II-Ce6 LSF in Figure 4B we find that there is a disconnect between the network of interactions linking rearrangements taking place in the CWxP motif (in the retinal-binding pocket) with a conserved intracellular pathway of intermolecular associations consisting of: a conserved network of internal water molecules (Nygaard et al., 2010), conserved residues in TM1 and TM2, and residues comprising the NPxxY motif in helix 7. This conserved pathway dynamically connects residues from the protein interior (in contact with the retinal) with residues in the cytoplasmic surface that are crucial for G_T_ binding. In Figure 4 and Figure S6, a comparison of the unbound and bound receptor structures from the MD simulation reveals that key water molecules involved with the activation pathway have been displaced in the Meta-II-Ce6 structure. The water-mediated H-bonding connection between Ala260 and Asn302 is disrupted in Meta-II-Ce6 and consequently, the connection between Trp265 in helix 6 and Asn302 in helix 7 is weakened. The modification in the conserved water-mediated network in Meta-II-Ce6 effects the rearrangement of H-bonding associations at the intracellular side of the receptor that form the G-protein binding site in the active state. Specifically, the rupture of the Ala260–Asn302 water-mediated H-bond linkage diminishes the correlation between the rotation “toggle” switch of the CWxP motif comprised of conserved residues Tyr268, Trp265, and Phe261 on helix 6 and the structural changes that take within the intracellular core of the receptor involving Met257, Tyr306, and Tyr223 that lead the “breaking” of the ionic-lock between Arg135 on helix 3 and Glu247 on helix 6. The ionic-lock connects the intracellular side of TM3 and TM6 in the active state and breaking of the lock is a crucial step in forming the G-protein binding site. In the MD simulations carried out in this study, we find evidence that the ionic-lock is not fully stabilized in the open form (broken) in the active-state of the Ce6-bound receptor (Figure S7).

#### Ce6-induced correlated fluctuations and the creation of an altered activation pathway in Meta-II-Ce6

**Figure 5:**
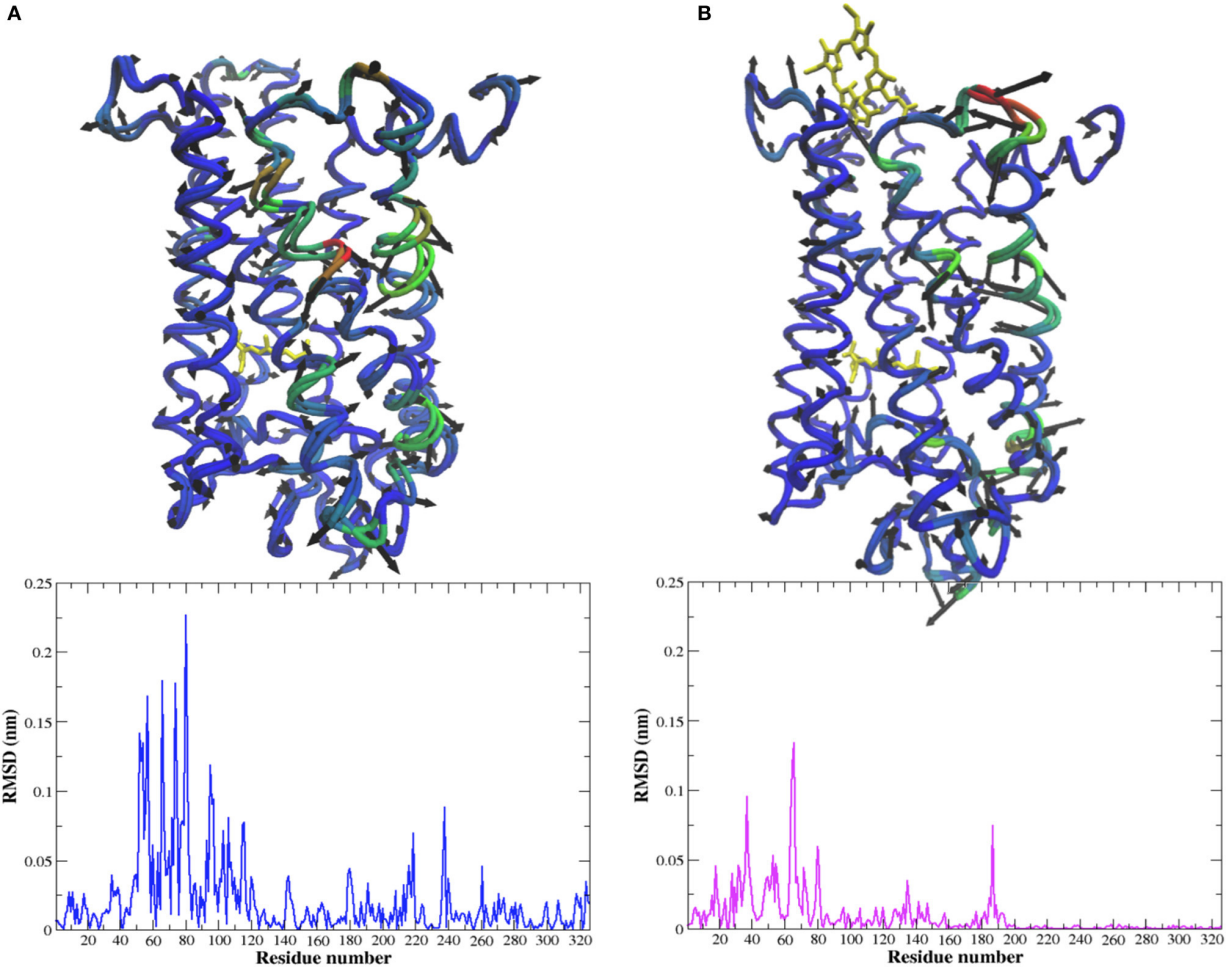
C-α representation of the dominant PCA mode (PCA1) and the corresponding per residue RMSD from the MD simulation of **(A)** Meta-II and **(B)** Meta-II-Ce6 where the areas in blue show regions with less (translational) mobility and the areas in red show regions with more (translational) mobility.

The reason for the disruption in the activation pathway becomes more apparent when analyzing the induced interactions and correlated motions that accompany Ce6 binding in Meta-II-Ce6. In Figure 5, we find that Ce6-induced correlated fluctuations introduce new LSFs in the vicinity of the receptor ligand-binding pocket. An illustration is provided in a comparison of the correlated dynamics in both receptors involving helix 4 near the ligand-binding pocket in Figures 4C,D. In the unbound, active-state receptor there is a strong correlation between residues in helix 4 with residues residing in the N-terminus region of the receptor. The helix 4–N-terminus correlated motion is associated with stabilizing the activation pathway that connects the dynamics taking place within the retinal pocket with the dynamics occurring in the cytoplasmic side of the receptor. For instance, in a previous computational study on squid rhodopsin it was revealed that Ala167 (in helix4) in addition to Ala304 (helix 7) and internal water molecules in the intracellular region of helix 7 are instrumental in creating a maximally connected H-bonding pathway that links the active-state retinal binding pocket with the cytoplasmic region (Bondar et al., 2011).

**Figure 6:**
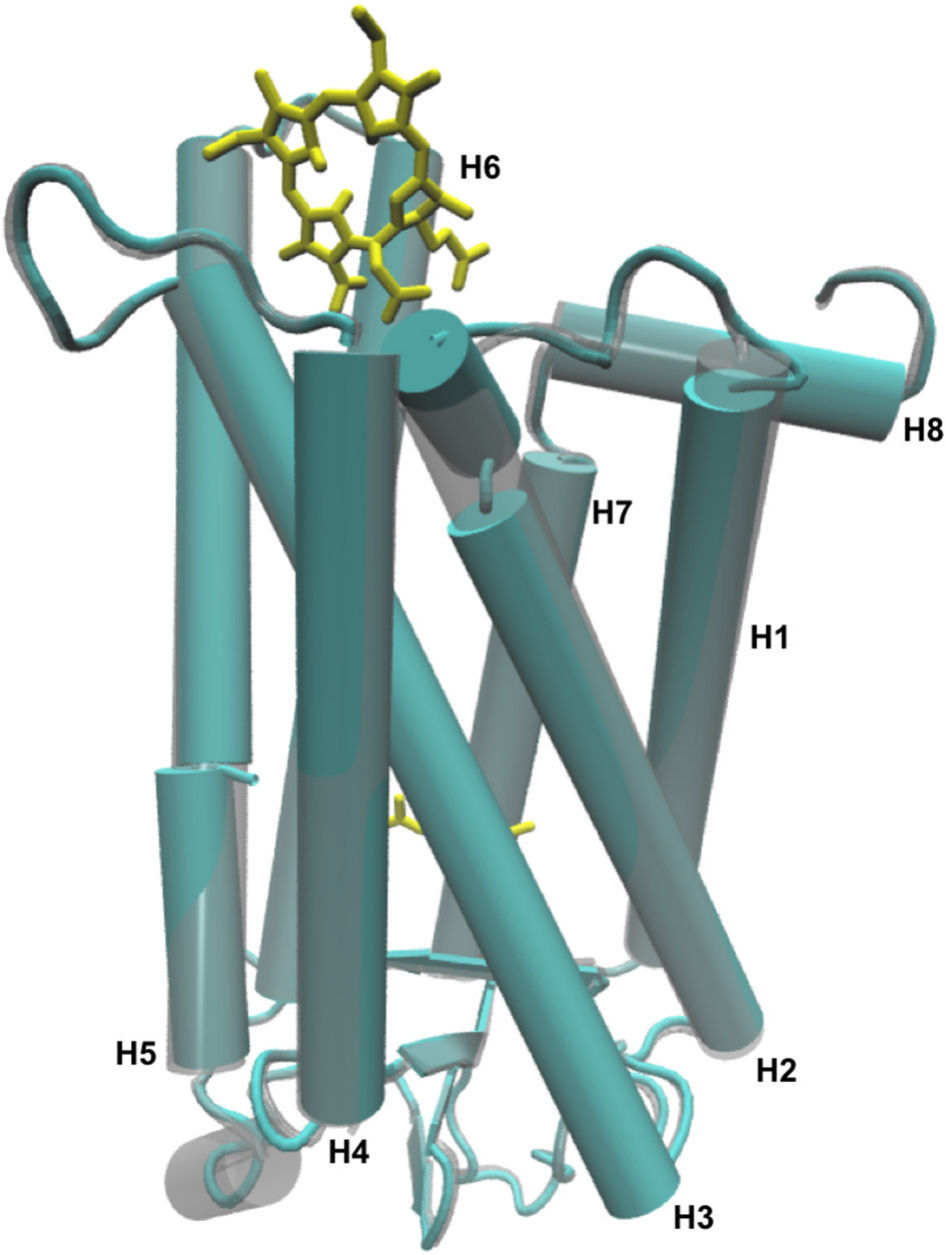
Overlap of a cartoon representation of the 3-D structure of Meta-II (cyan) and Meta-II-Ce6 (gray) from the MD simulations of Meta-II.

The absence of the (helix 4 - N-terminus) long-range connection in Meta-II-Ce6 suggests that the Ce6 bound receptor may support an alternate pathway for activation. For instance, in Figure 4A we observe that the binding of Ce6 excites new collective fluctuations in Meta-II that involve residues in CL3 near the Ce6-binding site that are coupled with collective fluctuations of residues in both EL2 and EL3. The Ce6-induced dynamical fluctuations have a prominent effect on the shape of the ligand-binding pocket and consequently, the allosteric interactions that determine the signal communication pathway from the ligand-binding site to the rest of the protein. Specifically, a comparison of the LSFs of the unbound and bound active-state receptor in Figures 4A,B reveals that Ce6-induced fluctuations disrupt long-range correlations between EL1 and residues that constitute the hydrophobic core of the receptor, particularly Ala117, Thr118, and Gly120–Glu122. In the unbound receptor, this long-range correlation is associated with maintaining the shape of ligand binding pocket (Figures 4A,C). The disruption EL1–receptor core correlated dynamics in Meta-II-Ce6 promotes a deformation of the ligand binding region that consequently allows residues surrounding β-ionone ring, such as Glu122 and Pro215 on helix 5, to shift closer to the retinal as well as to the extracellular side of helix 6. This shift simultaneously alters a stable network of polar interactions within the core of the receptor that connects the ligand-binding site (involving helices 3, 5, and 6) with the rest of the protein. Explicitly, the shift of helix 5 ligand-binding residues promotes a shift in the hydrophobic interactions involving Gly121 and Leu125 on helix 3 and Phe261 on helix 6. The adjustment of the hydrophobic packing interaction allows the intracellular side of the helix (helix 3) to move closer to helix 6. Further, the helix 3 shift is connected with a correlated fluctuation involving the motion of residues Phe261, Trp265, and Tyr268 on helix 6 with that of EL2, such that the movement of these residues promotes an overall tilt of the IC side of H6 and as a consequence also reduces the distance between TM3 and TM6 (Figure 6). The outcome is interhelical packing changes in the core of the molecule that shift TM2 and TM8 upward in the direction of the C-terminus of the receptor and tilts TM7 inward toward the hydrophobic core. Consequently, the altered ligand-binding cavity shape also supports a 7TM structure with a weakened but still associated Arg135–Glu247 “ionic-lock” (Figure S7) and a ligand-binding site with increased distance from EL2. In fact, the amplitude of the dynamical oscillations of the β_4_ loop of EL2 is far greater in the Ce6-bound receptor when compared with unbound Meta-II. This suggests that the increased distance between the binding pocket and the ECD in Meta-II-Ce6 provides an environment for the ligand that possess far less protection from hydrolysis and therefore a reduction in overall receptor stability when contrasted with the unbound receptor. Hence, the effect of the loss of long-range interaction connecting core residues with EL1 in Meta-II-Ce6 is the deformation of the ligand-binding pocket such that it modifies the intra-protein interactions that determine the shape of the G-protein binding site. And it also alters the contact interactions between the ligand-binding site and EL2 that are instrumental in forming the activation pathway in Meta-II.

The Ce6-induced deformation of the Meta-II ligand-binding pocket also supports a new set of associations that creates an alternative pathway for signal propagation when compared with the unbound receptor (Figure 4). The induced long-range correlated fluctuation extending from the ECD to ICD connect the dynamics taking place in the N-terminus of the receptor with fluctuations in EL3 and residues in helix 7. From an analysis of the Ce6-induced collective dynamics in Figure 4D, we find that the long-range interactions create large-scale torsional fluctuations in a cluster of hydroxyl residues in EL3 that separate helices 6 and 7. The residues with the largest contribution to the induced EL3 dynamics include Thr277, Ser281, and Pro285. The substantial increase in the magnitude of EL3 fluctuations subsequently alters the amplitude of rotational fluctuations of residues in helices 6 and 7 that line the retinal-binding pocket. These particular residues have previously been identified as having an important role in signal propagation and activation in rhodopsin and include Trp265, Pro267 - Ala271, Pro291, and Ala295. The large-scale induced-torsional fluctuations of these activation residues are instrumental in linking the dynamics of the ligand-binding site with the conserved pathway of residues forming the NPxxY motif. Their amplified motion compensates for the disruption in the intricate network of intermolecular interactions that form the activation pathway in Meta-II and in its place creates an anharmonic connection of associations that translate changes taking place in retinal binding region with the structural changes taking place in the ICD.

Together, the changes in the localized interactions induced by Ce6-binding result in conformational structural changes in Meta-II that promote and altered NPxxY motif and an altered activation pathway. Specifically, the binding of the allosteric modulator Ce6 supports a series of long-range interactions that result in structural differences in the TM3/TM6 distance of the active receptor as well as packing interactions between TM1/TM2/TM7 helices (Figure 6) when compared with the unbound receptor. These modifications support a narrowed G_T_ binding site that is likely less efficient in binding the G-protein and adopts a more structurally dynamic ligand-binding cavity that would have a direct effect on the thermal stability of the active-state receptor.

## Discussion

### Implications of Ce6 effects on rhodopsin for deep-sea ocean vision and spectral tuning

It has been proposed that Ce6 plays a role in modifying the receptor-chromophore interactions in rhodopsin that adapts the *Malacosteus niger* (*M. niger*) dragon fish visual system (Douglas et al., 1998, 1999, 2016; Kenaley et al., 2014). Ce6-induced modification in intra- and inter-protein interactions permit the *M. niger* species to emit far-red light from suborbital photophores, in addition to the blue bioluminescence that is normally emitted by deep-sea dragon fish. The suspected mechanism of the enhancement of long-wavelength sensitivity in the dragon fish is via spectral tuning (Kenaley et al., 2014). Spectral tuning is a molecular mechanism that shifts the optical properties of the retinal that regulate the absorbance maximum of the absorption of light. This can happen through evolutionary processes in which case, it involves a combination of adaptation and positive selection of key residues that directly interact with the retinal. Or alternatively, the tuning mechanism can be induced by adding small molecules that externally modulate opsin-retinal interactions (Washington et al., 2004, 2007; Isayama et al., 2006; Balem et al., 2009). Thus, the interaction of *specific* amino acids of the rod-opsin protein with the chromophore ultimately determines the peak absorbance (λ_max_) and therefore, also the spectral sensitivity of the pigment. It is well known that rhodopsin evolved from cone opsins (Imamoto and Shichida, 2014). Ancestral pigments were cone pigments and rod pigments evolved later in response to the necessity to function in dim light conditions. Rod opsins have a prolonged active-state. This is achieved by having high thermal stability, which promotes both slow thermal exchange and slow recovery. The advantage is that this allows for a longer signaling state, which both amplifies the response signal and also increases the overall sensitivity of the photoreceptor cell. Cone cells on the other hand, produce an active-state that is thermally unstable. In this respect, they also have a much faster signaling state and a faster rate of regeneration when contrasted with rod cells. These particular characteristics are best suited for operating in daylight conditions where the photoreceptor cells are under pressure to function under successive light stimuli.

**Figure 7:**
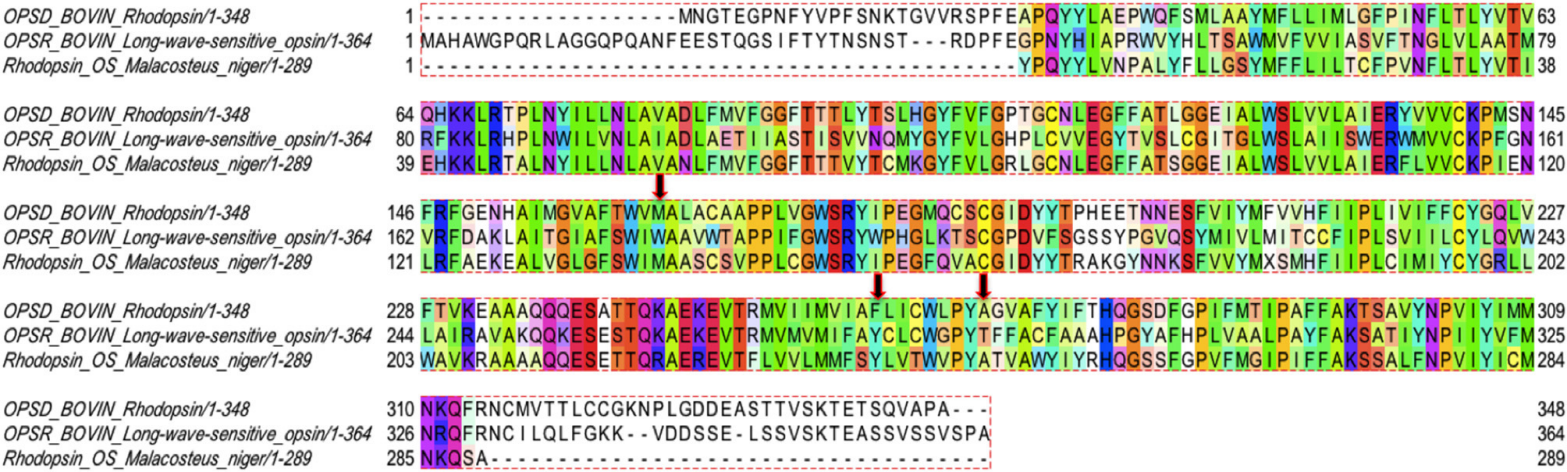
Sequence alignment of bovine rhodopsin (OPSD_BOVIN), long-wavelength bovine rhodopsin (OPSR_BOVIN), and *M. Niger* rhodopsin (Rhodopsin_OS_Malacosteus_niger) rendered with Taylor coloring.

We have previously proposed that Ce6 binding in rhodopsin takes place in the ICD of the receptor (Balem et al., 2009). The MD simulations and molecular docking predictions carried out in this investigation support this supposition. Thus, under this assumption, the Ce6-induced modulation of rhodopsin protein-ligand interactions would have to occur through an allosteric mechanism. However, the inferred mechanism of the Ce6-induced changes of rhodopsin functional dynamics from this study in many ways resonates more like an account of the *evolutionary* pattern of mutational changes that eventually transitioned ancestral cone cells into rod cells (Nathans, 1990; Lin et al., 1998; Rajamani et al., 2011; Imamoto and Shichida, 2014). In other words, the detected Ce6 modulation of rhodopsin functional properties mirrors the mechanism of spectral tuning brought about by mutational changes of specific amino acids in visual phototransduction evolution. For example, the observed “tuning” sites for Ce6-induced intermolecular and structural changes that we have detected in our experiments on bovine rhodopsin bound with Ce6 can be succinctly explained by contrasting them with the effects of well-known, critical mutational differences in the sequences of the two types of visual pigments. An illustration is given in Figure 7 were we have assembled an alignment of three different receptor sequences that include bovine rhodopsin, long-wavelength bovine opsin (i.e., red cone opsin), and *M. niger* rhodopsin. In the aligned sequences, bovine rhodopsin represents a characteristic rod opsin, whereas the long-wavelength and *M. niger* sequences represent a cone opsin and a red-shifted rod opsin respectively. The aligned sequences clearly highlight some of the most distinguishable differences of rhodopsin when compared with the other two spectrally shifted opsins (Figure 7). The alignment also provides further insight into the nature of the observed induced dynamical and structural changes that lead to differences in the detected spectral properties of Ce6-bound and unbound rhodopsin. But more importantly, it offers a deeper understanding about the evolution of critical long-range interactions involved in the transmission of the excitation signal from the binding site of the chromophore to the cytoplasmic surface. These long-range interactions in visual pigments have been extensively tuned over time to improve the sensitivity and stability of the more evolved rod receptors and their pathway of adaptation is tied with essential global changes in the molecular structure of the receptor that have been tailored to stabilize them.

Two of the most recognized critical residue changes in rod cells vs. that of cone cells are found at position 122 and 189 (in rhodopsin numbering) in the receptor sequences. The substitution of Ile for Glu at position 122 is known to be associated with a dramatic increase (blue-shift) in the wavelength of the photosensitivity of various cone cells when contrasted with rod cells (Lin et al., 1998; Lewis et al., 2006; Imamoto and Shichida, 2014). Similarly the substitution of Pro or Met for Ile at position 189 is related with increasing both the decay rate of the signaling state (Figures S9c, S10e,f) as well as the reconstitution rate of the chromophore in cone opsins (Janz et al., 2003; Imamoto and Shichida, 2014; Yanagawa et al., 2015). Despite this general knowledge, there is still the remaining challenge of developing an overall sense of how these single point mutations impact major properties of the receptor. For instance, in particular cases local perturbations of proteins have been shown to produce global changes that have an inclusive effect on protein function and/or stability. In rhodopsin we identify three such spots, where substitution of select residues have a significant destabilizing effect on both the global functional properties of the receptor and the nature of the signaling pathway of activation. It is particularly through these sites that Ce6 modulates the functional dynamics of the visual pigments through long-range, evolutionary-conserved interactions that establish communication between the chromophore and the rest of the receptor (Figure S6).

### Mechanisms of allostery in rhodopsin and the generality to other visual receptors

We have uncovered three specific allosteric sites in our investigation of Ce6-bound rhodopsin that are utilized to modulate the functional properties of the receptor. These sites are activated by Ce6 in the ICD of the receptor and are associated with moderating the coupling mechanism of the different structural components in the receptor that are necessary for signal activation and propagation in rhodopsin (Wolf and Grünewald, 2015). Particularly, through these allosteric sites, Ce6 modifies the signaling properties of rhodopsin by acting on distinct structural constraints that rearrange in response to activation and have a direct role in signal propagation.

### Coupling between the ligand-binding site and the ECD in Ce6-bound rhodopsin

One such allosteric site is Ala269 in rhodopsin. Ce6-induced interactions involving Ala269 have a direct role in altering the activation pathway in Meta-II rhodopsin (Figure 7) due to its role in stabilizing the ligand-binding site (Tsukamoto et al., 2010). For instance, Ce6 binding promotes reduced interactions between Ala269 and the β-ionone ring of the agonist. This change in residue-agonist binding affects the thermal stability of the entire receptor. The mechanism of the diminished receptor stability was deduced from an analysis of the Ce6-induced collective dynamics in Meta-II (Figures 4C,D). From the analysis we found that the Ce6-induced long-range interactions in rhodopsin create packing defects in the retinal-binding pocket that are allosterically transmitted to structurally conserved hydroxyl residues in EL3. The result is Ce6-induced, large-scale fluctuations in EL3 that directly modify the amplitude of the dynamics of residues in helices 6 and 7 that surround the retinal ligand. Thus, it is the increase in dynamics of these particular residues (in helix 6 and 7) that have a direct impact on the coupling mechanism that links signaling components (residues) in the ligand-binding site with those in the ECD. Their coupling is directly correlated with the basal activity of the receptor. The Ce6-induced dynamics in both dark-state and Meta-II rhodopsin foster a more structurally dynamic ligand-binding pocket that reduces the thermal stability of the receptor. We find evidence of the diminished stability in both our experimental (Figure 1D) and MD simulation analyses (Figure 5B) of Meta-II-Ce6.

Analogously, in the sequence alignment of the visual pigments in Figure 7 we notice a substitution of Thr269Ala in the cone pigment when compared with both rhodopsin sequences. The replacement of threonine for alanine at position 269 in the receptor structure would account for less stability in the ligand pocket of the cone pigment due to the introduction of a bulky side-chain that would disrupt the packing interactions of the β-ionone ring with the ligand-binding residue (Supplementary text, *MD simulation of Meta-II mutations*, Figures S9b,f, S10c,d, S11, S12).

Previous studies on rhodopsin have noted a relationship between ligand-binding pocket structural flexibility, the thermal stability of the receptor, and the receptor active state lifetime (Janz et al., 2003; Ala-Laurila et al., 2004; Yanagawa et al., 2015). They conjectured that the higher thermal stability of rhodopsin when contrasted with cone cells represents an evolutionarily adapted trade-off of photoreceptor speed for a high detection threshold. The residue difference (at position 269) in the sequence alignment of the cone sequence vs. that of the rod cells parallels the Ce6-induced changes that we have observed in dark-state and Meta-II rhodopsin. In both instances, instability in the ligand-bonding pocket is directly correlated with the thermal stability of the receptor. Furthermore, in on our own previous investigation on rhodopsin we have clearly established a correlation between the flexibility of the agonist ring with receptor conformational stability. Therefore, Ce6-binding in rhodopsin alters the coupling mechanism between the ECD and ligand-binding site, and accordingly also strongly modifies the thermal stability of the receptor. Unfortunately, it is not possible to precisely measure the Meta-II decay rates in the presence of Ce6 because Ce6 quenches tryptophan fluorescence (Balem et al., 2009) but there is compelling evidence from the analysis of the residual fluorescence that Meta-II does decay faster in the presence of Ce6 (Balem et al., 2009).

### Coupling between ligand-binding site and the IC region in Ce6-bound rhodopsin

Phe261 is another allosteric site that has been uncovered in our analyses of Ce6-bound rhodopsin. Phe261 (helix 6) is a highly conserved residue in the GPCR-A family. It, along with Gly121 (helix 3), forms a hydrophobic micro-domain in the interior of rhodopsin that rearranges during activation. The rearrangement of hydrophobic interactions between helix 3 and helix 6 are essential in translating retinal conformational changes that take place during activation into helical rearrangements in the intracellular region of the receptor that are propagated to the cytoplasmic surface. The mechanism of signal propagation from the ligand-binding site to the cytoplasmic region involves an intricate network of conserved H-bonding interactions that tightly couple the ligand-binding site with the IC region of the receptor (Fritze et al., 2003; Brown et al., 2009). This allosteric network of H-bonding interactions has been found in other class-A receptors, although the extent of coupling between the distinct components of the network varies amongst the different receptors. In our analyses on Ce6-bound rhodopsin, we find that the allosterically coupled motions leading from the ligand-binding site to the G-protein binding site is disrupted by the loss of conserved water molecules that comprise part of the NPxxY motif. Specifically, the Ce6-induced interactions in Meta-II lead to the loss of the conserved water molecule shared between Met257, Phe261, and Asn302 (Figure S6), which ultimately leads to hydrophobic packing defects in the protein interior and a less stable signaling pathway. The result is a weaker coupling between the allosteric components and a coarser translation of ligand-binding changes to the G-protein side of the receptor.

In the sequence alignment of the visual pigments in Figure 7, we observe a major change in the amino acid residue at position 261 in rhodopsin when compared with both the cone pigment and the *M. niger* rhodopsin sequence. Particularly, the substitution of Tyr261Phe in the cone pigment is known to lead to a much “looser” coupling between the ligand-binding site and the intracellular connections that lead to the G-protein binding region (Supplementary text, *MD simulation of Meta-II mutations*, Figures S9d,h, S10g). The replacement of tyrosine for phenylalanine at position 261 modifies the allosteric network of the cone pigment in a similar manner to what has been observed in the Ce6-induced disturbances of the intracellular signaling regions of rhodopsin (Figure 4, Figure S9g). It is well known that cone pigments maintain a more heterogeneous active-state after activation and less efficient G-protein activation when compared with rhodopsin (Imamoto and Shichida, 2014). The Tyr261Phe substitution has also been credited with a 10 nm blue-shift of the λ_max_ between cone and rod pigments (Chan et al., 1992), suggesting that the residue change is somehow connected with the modulation of the signaling network from the ligand-binding site. The stability and homogeneity of the active-state of rhodopsin is unique in the class-A receptors. The low basal activity and high photon detection efficiency of the receptor is attributed to the succinct coupling of the allosteric network of signaling components of the active-state.

### Coupling between the G-protein binding site and retinal ligand-binding site in Ce6-bound rhodopsin

We have also identified Met163 as an allosteric site in rhodopsin that has a central role in forming the G-protein binding site in the active-state protein. In our LSF analysis of the active-state of rhodopsin (Figures 4B,D) we found that long-range interactions between residues near Met163 and the N-terminus of the receptor are central in stabilizing the signaling network of interactions in Meta-II. Subsequently, in our analysis of Meta-II Ce6-induced long-range interactions (involving Met163) we determined that the Ce6-induced disrupted connection between helix 4 and the N-terminus allowed for the deformation of the ligand-binding pocket such that it modified the intra-protein interactions that determine the shape of the G-protein binding surface site (Figure 6). The disruption in the long-range interactions in Meta-II-Ce6 also accounted for the alteration in the contact interactions between the ligand-binding site and EL2 that are instrumental in forming the signaling pathway in the active-state in visual pigments.

Overall, we find that binding of Ce6 weakens conserved interactions that allosterically link the dynamics in the ligand-binding pocket with conformational fluctuations taking place at the receptor G-protein binding site. The decoupling of the distinct regions of the allosteric network result in a mixture of photo-intermediate conformations in the active-state of the receptor. For instance, interhelical distance fluctuations in the IC region of Meta-II-Ce6, due to instabilities in the conformational coupling of the structural elements of the receptor (Figure S7), alter the population of conformations in the active-state ensemble. An examination of the retinal torsional dynamics (Figure 3B) and the conformational ensemble dynamics (Figure S8) from the MD simulation of Meta-II reveals the interconversion between two dominant conformations in the active-state receptor when bound to Ce6. The dominant conformation (Meta-II_i_) has reduced distance between the IC region of helices 3 and 6 and an increased distance between the ligand-binding site and EL2, relative to the initial X-ray crystal structure of Meta-II used for the MD simulations. The secondary conformation (Meta-II_ii_) deviates only slightly from the initial Meta-II structure. The Meta-II_i_ conformation would presumably have a higher propensity for chromophore hydrolysis in the active-state and a conformation that is less efficient for G_T_ activation compared with Meta-II_ii_. Thus, the two photo-intermediate structures are associated with distinct intracellular signaling pathways that have ramifications on both the maximum G_T_ activity of the bound receptor as well as overall receptor thermal stability. In support of this conclusion, we have found in previous studies that the presence of Ce6 does indeed reduce G_T_ activation in a concentration dependent fashion (Balem et al., 2009).

Referring again to the sequence alignment of the visual pigments in Figure 7 we observe that the cone pigment has a tryptophan in position 163, whereas both rhodopsin sequences possess a methionine. The Trp163Met substitution in the cone pigment sequence introduces an amino acid with a large aromatic side-chain in a key position that would significantly alter the close packing interactions of helices 3–5 that support the active-state receptor structure (Supplementary text, *MD simulation of Meta-II mutations*, Figures S9a,e, S10a,b). Furthermore, the residue exchange would also considerably weaken crucial long-range allosteric connections between the ligand-binding site and IC residues (Figures S9a,e) associated with G-protein binding (when contrasted with Meta-II of the rod cell sequences). The effect would be a substantially weaker coupling between the allosteric components of the signaling pathway and a shift in the population of photo-intermediate states toward a conformation that overwhelming supports a reduced affinity for both the G-protein and the chromophore (retinal). It is widely recognized that cone pigments have a higher rate of thermal activation and much faster decay of the photo-activated pigment compared with rod pigments. The decay of Meta-II is the rate-limiting step for the termination of the light response in visual pigments and hence, one of the key factors that distinguishes rod cells from cone cells. For this reason, it has been conjectured that the differences in the two types of visual pigments are attributed to a *few* strategic amino acid changes that were acquired during the evolution of rod cells for dim light sensitivity. The long-lived G_T_ activation state with high efficiency is typical of rhodopsins, implying that rhodopsin acquired these specific characteristics over time to explicitly refine its function for single-photon detection sensitivity.

### Ce6-induced modulation of conserved allosteric sites in rhodopsin-like (GPCR-A) receptors and new strategies for drug design of GPCR allosteric modulators

**Figure 8:**
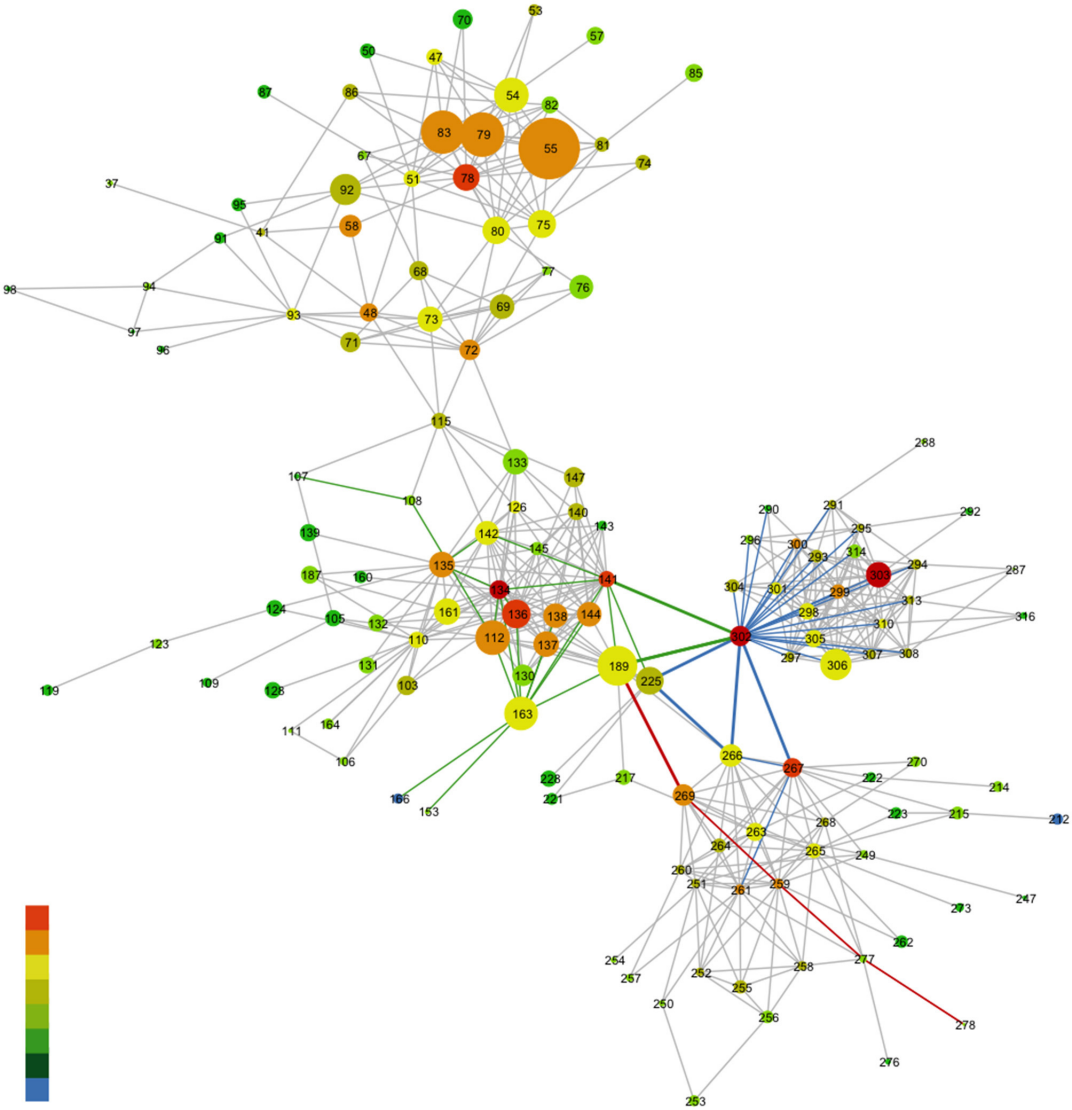
Network representation of the MSA of the rhodopsin-like family of proteins. Nodes represent a specific amino acid and gray edges represent the lines between the nodes. The rainbow coloring of the nodes characterizes the degree to which a given amino acid takes part in the MI network (denoting coevolution propensity). Red nodes have a high MI value and blue nodes a low MI value. The size of the nodes in this case corresponds to the conservation of the amino acid from the MSA. The colored edges highlight the associations between specific nodes. For more details refer to Supplementary Text, *Ce6 modulation of GPCR-A conserved allosteric sites in rhodopsin*. The reference structure for the MSA is bovine rhodopsin (opsd_bovin) with pdb ID 1u19.

Developing a clear understanding of how specific ligand modulators moderate topographically distinct allosteric sites in receptor families is one of the essential steps for the successful design of small-molecule allosteric drugs. The evolution of protein allosteric sites involves pairwise interactions that are responsible for the propagation of conformational changes from the ligand-binding site to a distal functional site. In visual receptors, these allosteric sites are linked with protein coevolved residue pairs (residues with evolutionary correlated mutational patterns) that have been selected for their regulatory properties and conformational ensemble dynamics that are directly involved with fine-tuning spectral sensitivity. In this work, we propose that the small-molecule allosteric modulator Ce6 binds specifically in the CP domain of rhodopsin. It acts by not only moderating the spectral sensitivity of rhodopsin, but on a higher level by modulating GPCR-A-wide conserved allosteric sites that facilitate coupling of receptor structural and functional domains (Figure 8, Figures S13, S14, and Supplementary text, *Ce6 modulation of GPCR-A conserved allosteric sites in rhodopsin*). Principally, we find that Ce6 binding in rhodopsin allosterically alters the receptor structure by mediating conserved long-range conformational fluctuations that modulate access to the retinal ligand-binding pocket. Why is this significant? Every currently known GPCR transmits a ligand-binding signal originating in the EC and/or TM domain to the CP domain via conformational fluctuations that take place in the TM domain. The CP domain is the site where the G-protein recognizes the active conformation of the receptors, and unlike other 7TM regions, the CP interface of the various GPCR-A receptors is relatively conserved. Therefore, these conserved residue positions at the CP interface form part of a signaling mechanism that allows the distinct receptors to bind and activate G-proteins by utilizing a common construction of coevolutionary residue pair interactions that form an allosteric regulatory network to the rest of the receptor. We conjecture that Ce6 modulates these same GPCR-A shared allosteric regulatory networks. For this reason, Ce6 may offer an initial stepping-stone for comprehending and creating small-molecule allosteric modulators that offer precise control of GPCR signaling pathways.

## Conclusions

In this investigation, we identify long-range conserved interactions in rhodopsin that are excited by the binding of an allosteric modulator (Ce6) in the cytoplasmic domain of the receptor. The excited structural fluctuations modify fundamental signaling processes that control receptor long-range interactions and are common in all Class-A GPCRs. In this specific case, we find that Ce6 stabilizes specific receptor conformations that alter the coupling mechanism between the distinct domains of the receptor and hence modify the GPCR signaling components that define rhodopsin function. These results provide deeper insight into the evolutionary coupled interactions in Class-A GPCRs that modulate the mechanism for coupling the ligand-binding site with both the ECD and G-protein binding sites and offers a foothold for elucidating how GPCR signaling pathways may be manipulated for allosteric drug discovery and design. Ce6 provides a clear illustration of a how small-molecule ligand modulator, associated with moderating the allosteric interaction networks and signaling pathways linked with spectral sensitivity in visual receptors, may also provide valuable insight into the (molecular) mechanisms that enhance or inhibit selective receptor pathways connected with evolutionary principles of allosteric regulation.

## Materials and methods

### Sample preparation

Rhodopsin samples were purified in detergent micelles composed of dodecyl maltoside (DM). The choice of DM as a detergent is justified because the conformational changes in rhodopsin in DM are virtually identical to those seen in liposomes (Kusnetzow et al., 2006). Rhodopsin samples were obtained through transient transfection or from stable cell lines. Transient transfection of COS-1 cells was carried out as described (Oprian et al., 1987), with the exception that the cells were harvested 72 h after transfection. Tetracycline inducible HEK293S stable cell lines were established as described previously (Reeves et al., 2002). Both types of cells were solubilized with 1% (w/v) DM for 1 h and the proteins were purified by 1D4 immuno-affinity chromatography in 0.05% DM as described (Hwa et al., 1999). Briefly, after solubilization of the cells, the suspension was centrifuged for 30 min at 35,000 rpm and 4°C. The supernatant was mixed with 1D4 Sepharose beads (approximate binding capacity of 1 μg rhodopsin/ μl of resin) for at least 6 h at 4°C. The resin was then washed with 50 bed volumes of 0.05% (w/v) DM in PBS followed by 10 bed volumes of 0.05% (w/v) DM in 2 mM Na_2_HPO_4_/NaH_2_PO_4_ (pH 6.0). WT and mutant proteins were eluted with 70 μM C-terminal nonapeptide (TETSQVAPA) in 0.05% (w/v) DM in 2 mM Na_2_HPO_4_/NaH_2_PO_4_ (pH 6.0). The initial concentration of the sample was determined by UV absorbance and subsequently diluted to a concentration of 200 μm in preparation for the THz spectroscopy experiments.

Ce6 was obtained from Frontier Scientific, Logan, UT. Ce6 was added to rhodopsin samples from a 100 mM DMSO stock solution or its dilutions, to a final concentration of 200 μm.

The rhodopsin samples used in the THz spectroscopy experiments were prepared by allotting 20 μL of the prepared sample onto a custom ordered diamond transmission window (Specac Co., United Kingdom). Excess water from the solution was initially removed by applying a low, steady flow of N_2_ gas over the sample droplet for approximately 3 min. The resulting sample was subsequently rehydrated by equilibrating the dried-off sample in a vacuum sealed container with the vapor pressure of a saturated salt solution at 20°C for a minimum of 3 days. A relative humidity (RH) of 97% was obtained from the vapor pressure of a saturated K_2_SO_4_ solution (Wexler and Hasegawa, 1954). The prepared rhodopsin sample was subsequently placed in a sealed transmission cell consisting of two diamond window substrates and a saturated salt solution was placed at the bottom of the cell to ensure that hydration was maintained throughout the experiment.

Illumination of the samples in all experiments was carried out with a Fiber-Lite DC 950 regulated illuminator by Dolan-Jenner industries.

### THz spectroscopy experiments

The dark-state rhodopsin experiments were performed under dim-red light conditions and photo-isomerization was triggered with visual light excitation. The THz spectroscopy experiments were carried out on a Jasco FTIR - 6000 series spectrometer. The protein sample spectra were collected with a liquid helium cooled bolometer in the 15–250 cm^−1^ spectral range. The 15–100 cm^−1^ THz spectra were collected with a 25-micron beam splitter while the data in the 100–250 cm^−1^ spectral region was collected with a 12-micron beam splitter. For each transmission measurement a 25 mm diameter region of the protein sample was illuminated with the THz beam to determine the absorbance. In the spectral measurements presented, each scan consists of 16 averaged scans and the infrared data was collected with a spectral resolution of 4 cm^−1^.

### Molecular dynamic simulations

#### MD simulation of dark-state rhodopsin and Meta-II

Each MD simulation consisted of a starting x-ray crystal structure taken from the PDB database. PDB structure 1 μ19 was used for the inactive (dark) state of rhodopsin and 3pxo was used for Meta II. In all simulations, the receptor was embedded in a hydrated lipid bilayer with all atoms represented explicitly. Specifically, the dark-state receptor and any resolved water molecules from the crystal structure were embedded in an equilibrated palmitoyloleoyl-phosphatidylcholine (POPC) bilayer consisting of 110 lipid molecules, and additional 7400 water molecules, and 100 mM NaCl (to neutralize the net charge of the system). The membrane system was built with the use of the g_membed tool in Gromacs. All titratable groups in the receptor were considered to be charged (Fahmy et al., 1993). The exceptions were Asp83 and Glu122, which were both neutral in both the dark-state and Meta II MD simulations. Also for the dark-state MD simulation, the Schiff base was protonated whereas Glu113 was deprotonated. For the Meta II simulation both the Schiff base and Glu113 were set to neutral. The active state receptor combined with the structural waters from the crystal structure was prepared in a similar manner to that of the dark-state. MD simulations were performed at 300 K using the Gromacs package (www.gromacs.org) version 5.0. The GROMOS96 43a2 force field parameters were utilized for the protein and the Berger lipid parameters were used for the lipid component of the membrane protein (Berger et al., 1997). The SPC water model was used for hydration and the ground-state retinal parameters (Bondar et al., 2011) for both the 11-*cis* and *all-trans* retinal chromophore were obtained from the Bondar group.

In the rhodopsin simulations, energy minimizations were initially carried out to reduce the number of unfavorable contacts between added solvent molecules and the receptor using a steepest descent method to a convergence tolerance of 0.001 kJ mol^−1^. The energy minimization was followed by a MD run with constraints for 200 ps in which an isotropic force constant of 100 kJ mol^−1^ nm^−1^ was used on the protein and lipid atoms. During the restrained dynamics simulation, the temperature and pressure of the system were kept constant by weak coupling to a modified velocity rescaled Berendsen temperature (Berendsen et al., 1984) and pressure baths and in all cases the protein, lipid, water, and ions were coupled to the temperature and pressure baths separately. The output conformation from the MD simulation with constraints was used as the starting conformation for an initial 200 ns equilibrium MD simulation.

Six subsequent simulations were conducted where randomized conformations from the last 10 ns of the equilibrium simulations were used as starting point conformations for each distinct simulation. These subsequent simulations were carried out for an additional 500 ns and were eventually used to assess the picosecond time scale fluctuations in the receptor systems. The final simulations were carried out with a 1 fs time step where the bonds between the hydrogen and the other heavier atoms were restrained to their equilibrium values with the linear constraints (LINCS) algorithm (Hess et al., 1997). Particle mesh Ewald (PME) method (Essmann et al., 1995) was used to calculate the long-range electrostatic interactions in the simulation and was used with a real-space cutoff of 1.0 nm, a fourth order B-spline interpolation and a minimum grid spacing of 0.14 nm.

MD simulations of Meta-II mutants (single and double mutations) were carried out by creating a residue mutation(s) with DUET (http://biosig.unimelb.edu.au/duet/), a web server for studying missense mutations in proteins. Minimization and production run MD simulations on the mutated receptors were carried out in a manner analogous to that described for WT rhodopsin.

Trajectory snapshots, each containing a record of the atom positions and velocities at a particular instant in time, were saved every 100 fs during the production simulations.

### Modeling and docking studies

Ce6 docking studies on dark-state rhodopsin and Meta-II were performed using the AutoDock 3.0 program (Goodsell et al., 1996). Modeling and docking studies of the G_t_ C-terminal high affinity peptide were performed using the MODELER (Sali et al., 1995; Fiser and Sali, 2003) and ClusPro docking software (Comeau et al., 2004), respectively. SwissDock (http://swissdock.vital-it.ch/) web services (Grosdidier et al., 2011a,b) were used as a secondary verification method to determine the binding sites of Ce6 on rhodopsin. In this case, we assumed a blind docking estimate of the binding modes comprising the most favorable energies in the docking calculations. The highest populated cluster of Ce6-rhodopsin predicted a conformation with Ce6 bound to rhodopsin near the retinal-binding site, whereas the lowest energy cluster favored a ligand orientation in the cytoplasmic region with binding specifically favored close to residues in CL2. The output of the docking results were visualized with the UCSF Chimera (http://www.cgl.ucsf.edu/chimera/) molecular modeling system (Pettersen et al., 2004). Subsequent MD simulations for dark-state rhodopsin with Ce6 and Meta-II-Ce6 were carried out in an analogous manner to what has been described for the unbound receptors in the previous section. The simulations comprising Ce6 at the CP interface were the only conformations found to stably bind the allosteric ligand (Ce6) with a consistent binding site. Ce6 binds weakly in the CP region of inactive-state rhodopsin with an itinerant path limited to regions near the receptor C-terminus. In Meta-II, Ce6 has a clear, preferred binding site involving residues comprising CL2. The simulations containing Ce6 bound to regions near the receptor *retinal-binding site* found the ligand migrating away from the receptor within the first 10 ns in *all* MD simulations conducted and were not considered further for analyses conducted in this study. Using the RMSD based on the distances between structures, the g_cluster algorithim in gromacs was used to determine the range of accessible conformations in the Meta-II-Ce6 simulations (Palczewski et al., 2000; Menon et al., 2001; Ahuja et al., 2009).

### Principal component analysis (PCA)

Principal component analysis or PCA is generally employed to detect correlations in large data sets. In MD simulations, the method can be utilized to reveal the most important motions in proteins. In this study, principal component analyses (PCAs) were carried out by diagonalzing the covariance matrix *C*_*ij* = 〈(_*x*__*i*_−〈*x*_*i*_〉)(*x*_*j*_−〈*x*_*j*_〉)〉_, where *x* denotes protein atomic positions in the 3*N*-dimensional conformational space and the angular brackets represent the averages over the MD trajectory. Translational and rotational motions were removed by a least squares fitting to a reference structure. The eigenvectors of ***C*** were determined by diagonalization with an orthonormal transformation matrix. The resulting eigenvectors from the transformation were used to determine the PCA modes with eigenvalues (λ) equivalent to the variance in the direction of the corresponding eigenvector. The MD trajectory was projected onto the principal modes to determine the principal components. The eigenvalues λ_*i*_ of the principal components denote the mean square fluctuation of the principal component *i* and are arranged so that λ_1_ ≥ λ_2_ ≥ … ≥ λ_3*N*_. Using this arrangement, the trajectories were filtered along the first principal component to analyze the collective dynamics taking place within the protein. The cosine content of the PCA modes presented were found to be less than 0.001.

### Determining rhodopsin-Ce6 ligand binding affinity from MD simulation with an alchemical pathway method

The ligand-binding affinity calculations were performed with Gromacs. The dissociation of the ligand from the receptor to determine the free energy of binding was determined by decoupling the van der Waal and Coulombic interactions of the ligand from the receptor by using an alchemical pathway (Boyce et al., 2009). Specifically, the alchemical pathway begins from the most accessible ligand-receptor conformation from the production run (unrestrained) MD simulation described previously. Subsequently, a harmonic restraint is added to the ligand to “pull” it away from the receptor ligand-binding site over a series of restrained conformational intermediates. The set of restraints used for pulling the ligand away from the receptor are described by one distance, two angles, and three dihedral harmonic potentials. Particularly, the distance restraint is defined by bonded terms between the ligand and the protein. In (dark-state) rhodopsin the distant restraint is described by the hydrogen-bond shared between the Pro347 backbone oxygen and hydrogen atom on one of the ligand pyrrole rings. For Meta-II the distance restraint is defined by a hydrogen-bonding interaction between the Arg147 side-chain and the nitrogen atom on the Ce6 pyrroline ring. The ligand restraints were comprised of 13 distributed λ values (or windows) of 0.0, 0.01, 0.025, 0.05, 0.075, 0.1, 0.15, 0.2, 0.25, 0.3, 0.5, 0.75, and 1.0. Initially, all the intermediate states were equilibrated. Each window was energy minimized using the steepest descent algorithm. The receptor-ligand system was subsequently simulated for an additional 1.0 ns in the canonical ensemble with harmonic position restraints applied to the heavy atoms (of the receptor and ligand) with a force constant of 1000 kJ mol^−1^ nm^−2^. Langevin dynamics (Goga et al., 2012) was used to set the reference temperature of the system to 300 K. Then, a 1 ns position restrained run in the isothermal–isobaric ensemble was conducted by using the Berendsen coupling algorithm for a target pressure of 1 atm. Finally, the production runs were carried out using Langevin dynamics for 30 ns. In all simulations, the particle mesh Ewald (PME) algorithm was used for electrostatic interactions with a real space cutoff of 1.2 nm, a spline order of 6, a relative tolerance of 10^−6^, and a Fourier spacing of 0.10 nm. The Verlet cutoff scheme was used with a van der Waals cut-off of 1.2 nm and a tolerance of 0.005 kJ mol^−1^ ps^−1^. A long-range dispersion correction for energy and pressure was employed and an *additional* long-range dispersion correction (EXP-LR) (Shirts and Chodera, 2008) was also utilized to compensate for using a van der Waals cutoff in a non-isotropic receptor-ligand system. Free energies were computed with the Bennett Acceptance Ratio (BAR) using the g_bar tool in Gromacs. The final free energy value was determined from the mean of all the free energy values from the separate simulations and the error computed from the standard deviation of the separate runs.

### Localized structural fluctuations (LSFs)

Localized structural fluctuations (LSFs) are local relaxations that reflect specific intramolecular and intermolecular induced protein fluctuations. LSFs have also been hypothesized to form the basis of allosteric signal propagation in proteins (Daily and Gray, 2007). The localized structural fluctuations from the MD simulations carried out on rhodopsin were calculated with the method of Pandini et al. (2013) that utilizes a structural alphabet (SA) to define protein local structural fluctuations that are described by a set of 25 canonical states composed of four-residue protein fragments. The four-residue fragments define the most probable protein local, conformational fluctuations in the protein 3-D structure. Structural correlations between local conformational changes of two protein fragments were calculated as a positional mutual information (MI) matrix between two column positions in the SA alignment.

### Multiple sequence alignment (MSA), residue conservation, and coevolution analysis

Multiple sequence alignment (MSA) is a powerful computational tool for uncovering the long-term evolutionary record of a protein family. The interdependence of evolutionary history or coevolution, which can be obtained from MSAs, is also used in this study to predict intermolecular communication between residue pairs and therefore, allosteric coupling that is related to protein functionality. The Class A Rhodopsin-like MSA data set was retrieved from the GPCR database (http://gpcrdb.org/). The reference sequence and structure were set as opsd_bovin with the PDB code 1 μ19. The conservation and co-evolutionary analyses on the rhodopsin-like family of sequences were carried out with the MISTIC server (http://mistic.leloir.org.ar/index.php). The mutual information score as implemented in MISTIC is calculated between pairs of columns in the MSA. The frequency for each amino acid pair is calculated using sequence weighting along with low count corrections and compared with the expected frequency. It is assumed that mutations between amino acids are uncorrelated. The MI score is calculated as a weighted sum of the log ratios between the observed and expected amino acid pair frequencies. The MI scores were translated into MI *z*-scores by comparing the MI values for each pair of positions with a distribution of prediction scores obtained from a large set of randomized MSAs (Buslje et al., 2009). The *z*-score is then calculated as the number of standard deviations that the observed MI value falls above the mean value obtained from the randomized MSAs. A *z*-score threshold of 6.5 describes a sensitivity of 0.4 and a specificity of 0.95. MISTIC lists every MI value between two residues with a value ≥6.5.

### Visualization of networks

Only the top 500 MI network links and nodes from the MSA were visualized. The position of residues in the two-dimensional MSA networks was computed with a combination of classical scaling and stress minimization (Brandes and Pich, 2009). And the network groupings were based on community detection resulting from modularity maximization (Newman, 2006). The network layout and grouping were calculated using the software tool Visone (http://visone.info/).

## Author contributions

KW performed and analyzed the THz experimental measurements and conducted and analyzed the MD simulations and the MD simulation calculations. KW wrote the manuscript. JP carried out the network analyses on the MSA datasets and created the 2-D and 3-D LSF network visualizations from the MD simulations. JK-S supervised the rhodopsin sample preparation, discussed the manuscript with KW and edited the text of the manuscript.

### Conflict of interest statement

The authors declare that the research was conducted in the absence of any commercial or financial relationships that could be construed as a potential conflict of interest.
