# Supplementary material for: Chlorophyll-Derivative Modulation of Rhodopsin Signaling Properties through Evolutionarily Conserved Interaction Pathways
Source: Front Mol Biosci. 2017 Dec 12;4:85. doi: 10.3389/fmolb.2017.00085 (PMC5733091; doi:10.3389/fmolb.2017.00085)
Supplement: Supplementary file 1 [file Image1.PDF]

# Chlorophyll-derivative modulation of rhodopsin signaling properties through evolutionarily conserved interaction pathways

Kristina N. Woods[1*], Jürgen Pfeffer[2], and Judith Klein-Seetharaman[3]

[1] *Lehrstuhl für BioMolekulare Optik, Ludwig-Maximilians-Universität, 80538 München, Germany*

[2]*Technical University of Munich, Bavarian School of Public Policy, 80333 München, Germany*

[3]*Warwick Medical School, University of Warwick, Coventry CV4 7AL, UK*

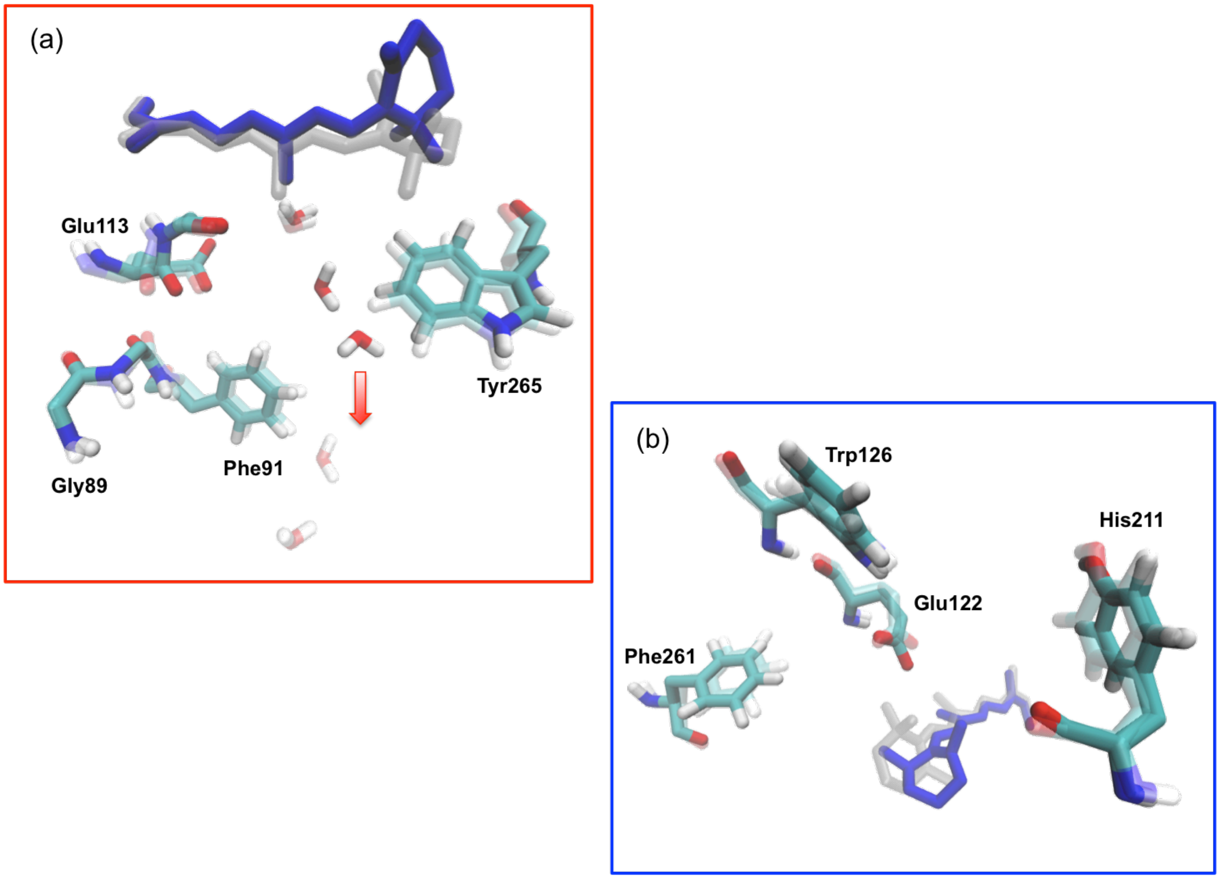


**Figure S1**: (a) MD simulation snapshot of ligand-binding pocket residues and *11-cis* retinal in dark-state and dark-state-Ce6 (transparent) rhodopsin depicting the expulsion of conserved water molecules in the Ce6 bound receptor that alter both the binding pocket shape and inter-protein interactions in the ligand-pocket interior. (b) MD simulation snapshot of residues in close-proximity to the retinal β-ionone ring in dark-state and dark-state-Ce6 rhodopsin (transparent) showing how induced-correlated fluctuations due to Ce6 binding cause H-bonding changes in retinal-protein interactions in the Ce6-bound receptor.

* Corresponding author: kristina.woods@physik.lmu.de

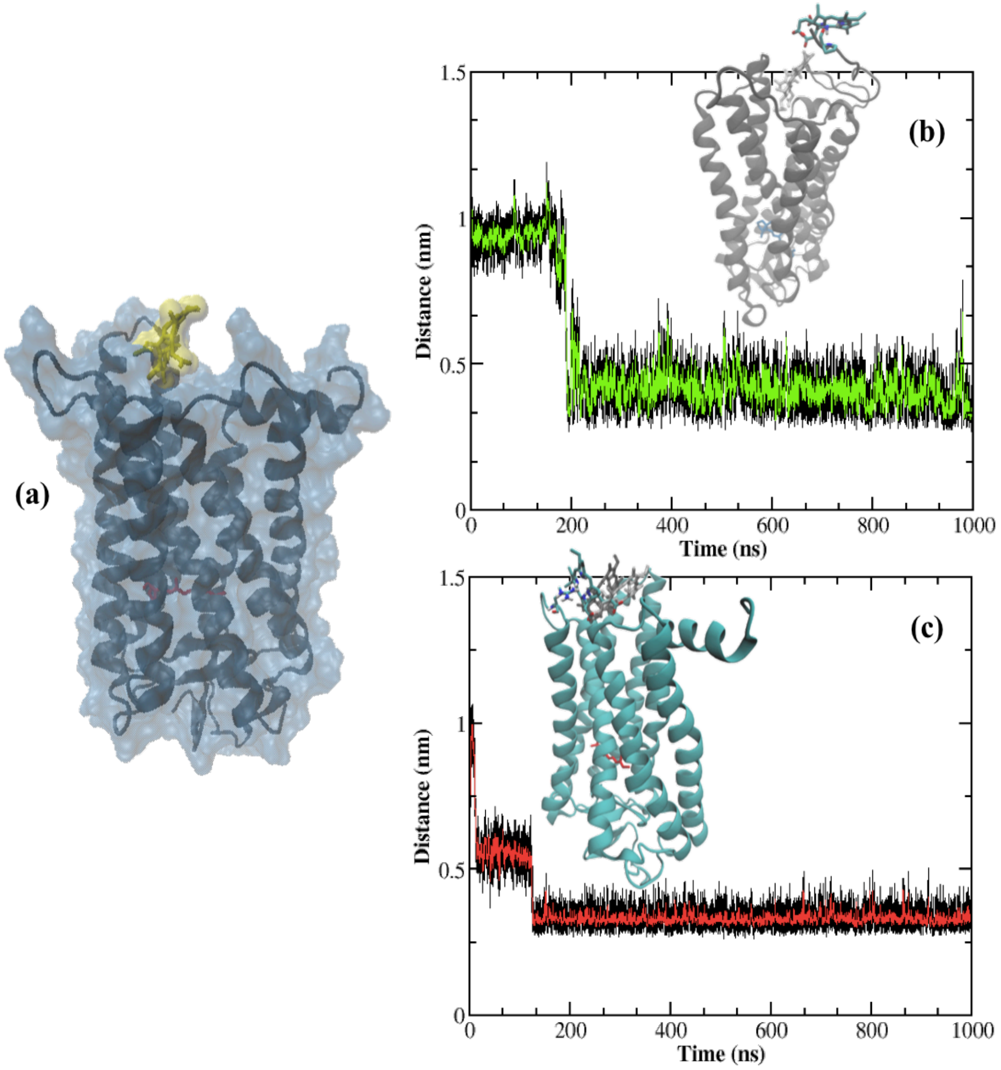


**Figure S2:** (**a**) The initial low-energy conformation of Ce6 from docking. (**b**) The distance of Ce6 to Pro347 as a function of time from the MD simulation of (dark-state) rhodopsin bound with Ce6. The green, solid line is a moving average of 100 steps. The inset shows a C-α representation of rhodopsin with the initial position of Ce6 shown in transparent gray and the final position shown with atom specific colors. (**c**) The distance as a function of time of Ce6 to Arg147 in the MD simulation of Meta-II-Ce6. The red, solid line is a moving average of 100 steps. The inset shows a C-α representation of rhodopsin with the initial positions of Ce6 shown in transparent white, a secondary position in transparent gray, and the final position shown with atom specific colors.

## Supplementary text, *Ce6 ligand-binding affinity in rhodopsin*

The initial docking position of Ce6 in rhodopsin is in the IC cleft almost equidistant from CL1 and CL2 (Figure S2a). During the course of the MD simulation, Ce6 interaction with the dark-state receptor and with Meta-II differs significantly (Figures S2b and S2c). In dark-state rhodopsin, Ce6 weakly binds to Pro347 on the receptor C-terminus. The Pro347 – Ce6 interaction leads to a local unwinding of the receptor unstructured C-terminus/Helix 8 region (1) (Figure S2b). In contrast, Ce6 migrates toward CL2 in Meta-II where it is stably held in the IC cleft by a H-bonding interaction with Arg147 (Figure

S2c).  A subsequent ligand-binding affinity study computed using an alchemical pathway method (2), reveals a free energy of binding of -3.72 ± 0.32 kcal/mol ($K_D$ = 2.0 mM) for the dark-state receptor and -4.32 ± 0.46 kcal/mol ($K_D$ = 0.75 mM) for Meta-II in the Ce6-bound MD simulations.  The computational results are in line with our experimental Ce6 ligand-binding affinity (fluorescence) measurements which determined a value of 2.6 mM for dark-state rhodopsin and 0.82 mM for Meta-II.  It is interesting to note that in the process of decoupling the ligand from the receptor to determine the free energy of binding from the MD simulations, we also observe that Ce6 dissociation from Meta-II leads to a global conformational change that resembles the conformational adjustments (3) that take place during light activation (Figure S3b).  Particularly, we observe rotation of helix 6 as well as a change in tilt in helices 4 and 5 due to rearrangements in EL2. Interestingly, the most prominent changes in structure involve helix 8 and CL2 in the decoupled Ce6-rhodopsin interaction.  It is likely that the large displacement of both groups (helix 8 and CL2) are associated with regulating the binding affinity on the cytoplasmic surface of the receptor (4).  The decoupling of Ce6 from the dark-state receptor results in subtler conformational rearrangements (Figure S3a).

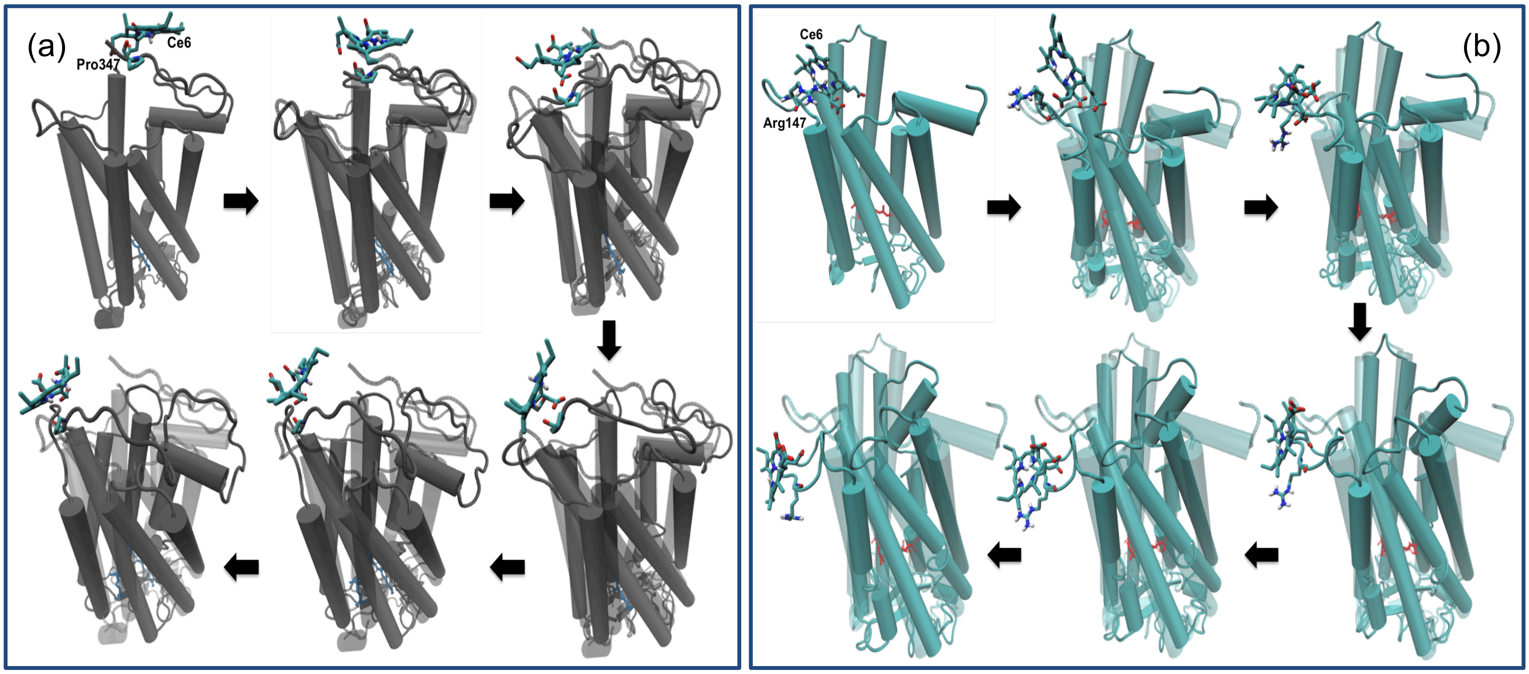


**Figure S3:** **(a)** Conformational states depicting the progression of the decoupling of Ce6 from (dark-state) rhodopsin that occur from conducting separate simulations with different restraining values that decouple coulombic and then Lennard-Jones interactions from the receptor-ligand system.  **(b)** Like **(a)** but involving the decoupling of Ce6 from Meta-II.

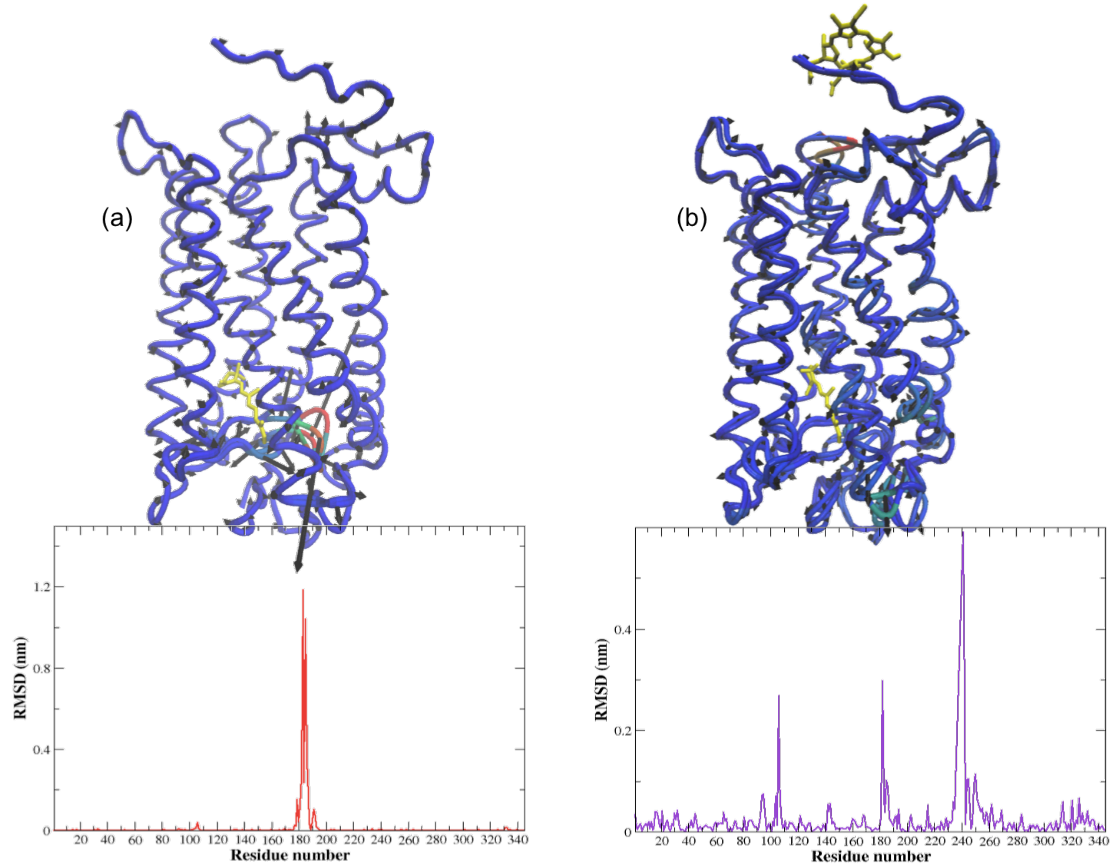


**Figure S4:** C-α representation of the dominant PCA mode (PCA1) and the corresponding per residue RMSD from the MD simulation of **(a)** dark-state rhodopsin and **(b)** dark-state-Ce6 where the areas colored in blue depict regions with less mobility and the areas in red show regions with more mobility.

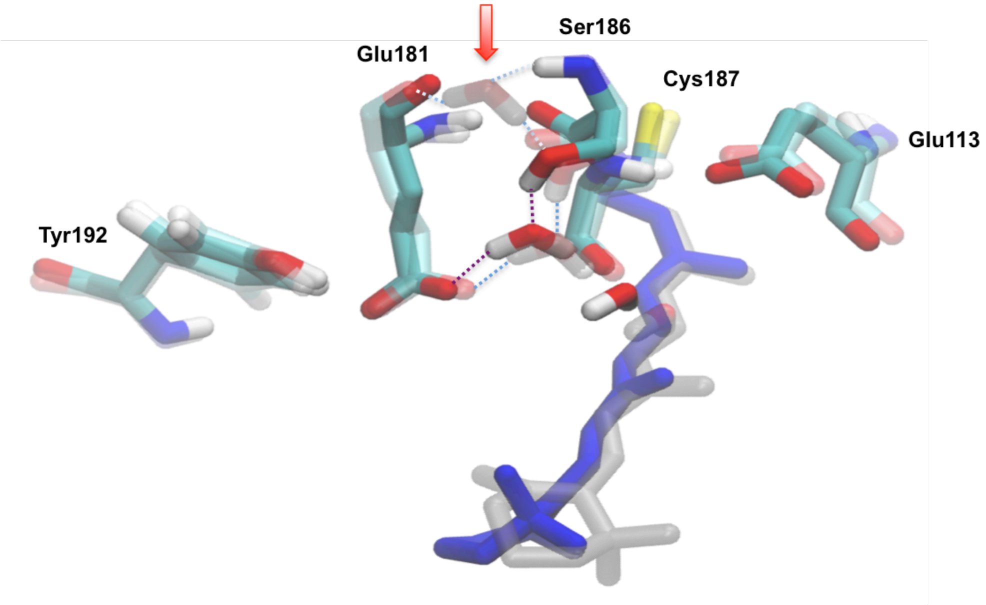


**Figure S5:** MD simulation snapshot of EL2 residues in dark-state and dark-state-Ce6 (transparent) rhodopsin illustrating how the addition of a water molecule in the EC region of the ligand-binding pocket of the Ce6-bound receptor alters the binding-pocket thermal stability and structural flexibility.

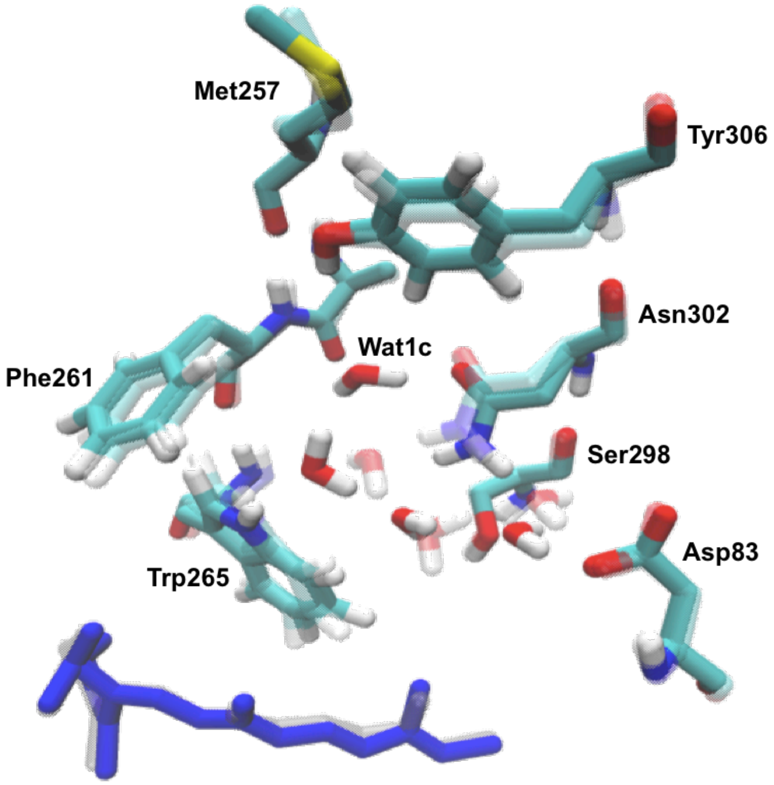


**Figure S6:** MD simulation snapshot of Meta-II and Meta-II-Ce6 (transparent) illustrating how loss of a conserved water molecule (Wat1c) in the intracellular region of the Ce6-bound receptor modifies the activation pathway of the excited state receptor that leads from the ligand-binding pocket to the receptor cytoplasmic surface.

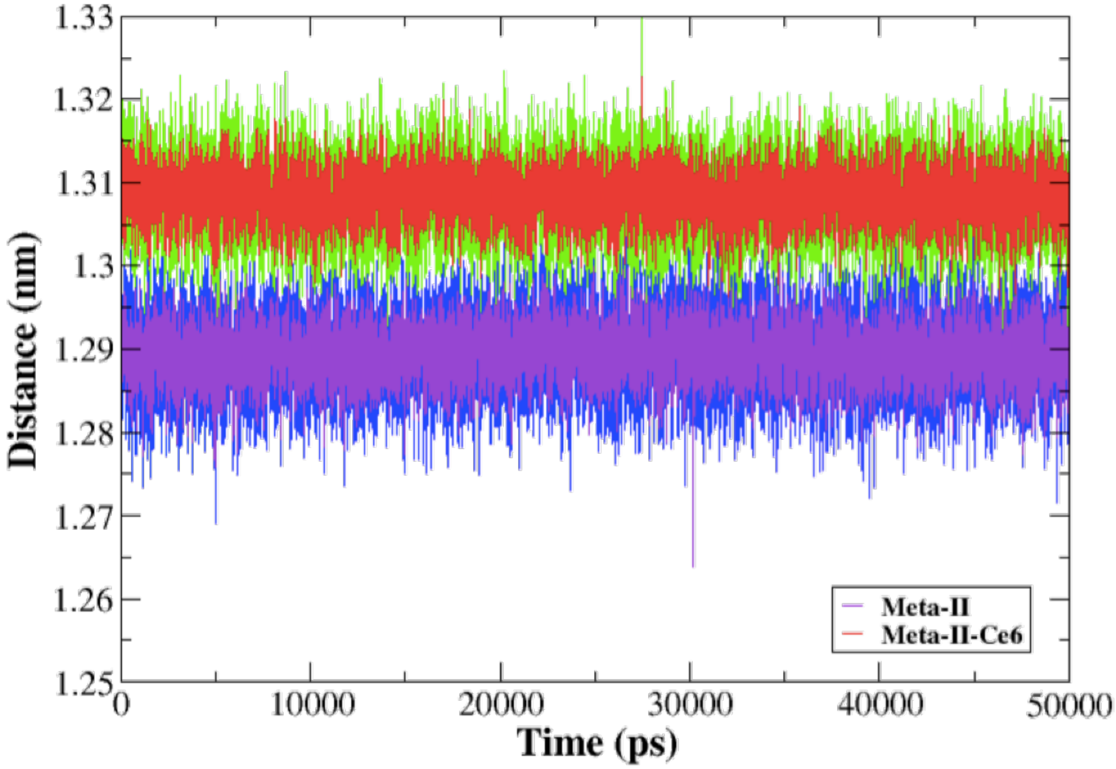


**Figure S7:** Distance of Tyr223 – Arg135 interaction as a function of time from the 1st 50 ns of the production runs of the MD simulation of Meta-II (purple line) and Meta-II-Ce6 (red line). The intact association of Arg135 – Glu247 in Meta-II-Ce6 leads to the consequent weakening of constraints that maintain the active-state orientation of the receptor.

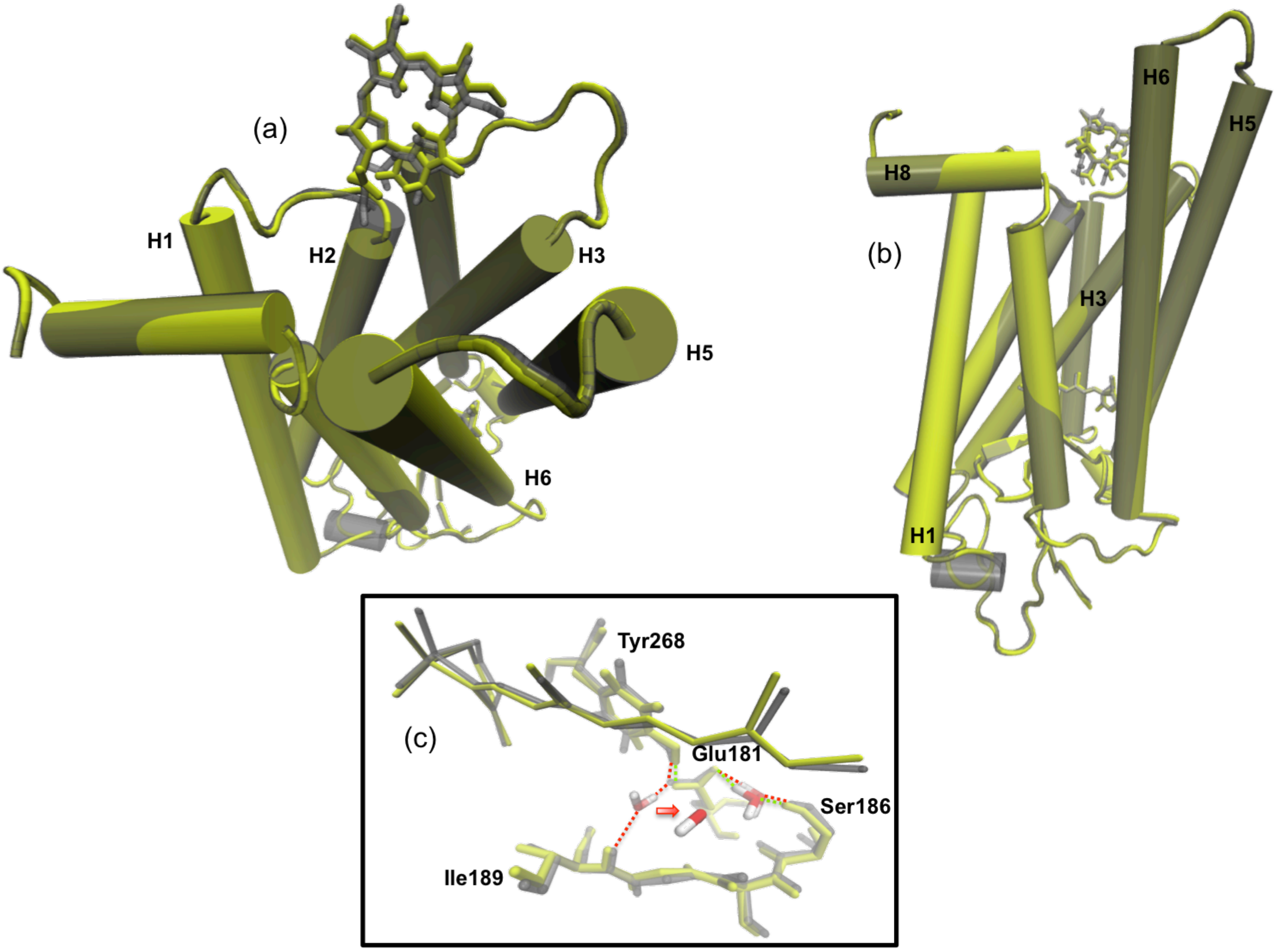


**Figure S8: (a)** Top view and **(b)** side view of the two dominant photo-intermediate states revealed from a structural ensemble dynamics analysis of the MD simulation of Meta-II-Ce6. The principal conformations consisted of a conformational state with lower affinity for both the agonist and the G-protein (grey) and a conformational state that resembled the starting crystal structure of Meta-II (yellow). In the manuscript, the structural states are referred to as Meta-II$_i$ and Meta-II$_{ii}$, respectively. **(c)** Depicts H-bonding changes in Meta-II$_i$ that come about through the transient influx of a water molecule in the EC region of the ligand-binding pocket, creating an increased distance between the ligand-binding site and EL2 when contrasted with Meta-II$_{ii}$.

**Supplementary text, *MD simulation of Meta-II mutations.*** From our MD simulations on single and double mutations on Meta-II rhodopsin, we determine that many of the single mutations that were identified as having a global effect on the receptor structural and dynamical properties are also associated with the prospective receptor loss of function. Surprisingly, when multiple (more than one) globally significant mutation is introduced, the receptor characteristics are considerably altered yet, for the most part, the functional properties are retained.

*Single point mutation and loss of functional properties in Meta-II rhodopsin*

***M163W***. Met163 in rhodopsin is in a sensitive to the environment around the retinal that is altered during retinal isomerization and receptor activation. Met163 is instrumental in forming the intra-protein interactions that induce helical rearrangement during the transition to the active-state intermediate Meta-II. The mutation of tryptophan for methionine in position 163 disrupts (hinders) the tight interhelical coupling in the region around the ligand-binding pocket such that the intracellular packing interactions involving helices 3 – 6 and the hydrogen-bonding interactions involving helices 3 and 5 are not displaced during the transition from the dark- to active-state conformation. Specifically, we observe from the LSF (Figure S9a) and from structural fluctuations identified from the MD simulation that the single-point mutation supports an intra-helical hydrogen-bond between Glu122 and Trp126 on helix 3 (Figure S10a). The Glu122-Trp126 interaction hinders the Meta-II H-bonding interaction between Glu122 and His211. The Glu122-His211H-bond is important for stabilizing the active-state conformation of the receptor by supporting the outward rotation of the intracellular end of TM helix H6, which functions to expose the G protein-binding site during activation. We also observe in the M163W Meta-II mutant that the hydrophobic packing interaction between helices 3 and 6 involving Gly121, Leu125, Phe261, and Trp265 is maintained in a conformation that resembles interhelical contacts formed in the inactive-state receptor (Figures S9a and S10b). The rearrangement of hydrophobic packing interactions in the receptor core is central in the isomerization of the retinal (moving helix5 closer to the 🚲-ionone ring to make space for the change in the retinal orientation) in addition to significant changes in intra-protein interactions that are essential for the motion of Trp265. The change in the position of Trp265 is required for the receptor to adopt an active-state conformation. The rearrangement of packing interactions in the receptor core is also coupled with the displacement of EL2 away from the retinal binding pocket in Meta-II. For instance, the retinal C19 methyl group in the inactive-state of rhodopsin is tightly constrained in the retinal-binding site and is packed against Thr118 (helix 3), Ile189 and Tyr191 (EL2) and Trp268 (helix 6). The loss of contacts between the C19 methyl group and Tyr191 and Tyr268 in Meta-II facilitates the modification of hydrophobic interactions near the retinal-binding site in the active-state that consequently also allows the Trp265 side-chain to rotate toward the extracellular surface. The release of Trp265 allows the intracellular end of helix 6 to rotate outward and a new contact to form between Arg135 and Met257 by breaking the Arg135-Glu247 ionic-lock between helix 3 and helix 6.

From our analysis of the M163W Meta-II MD simulation we find that mutation-induced interactions of the active-state receptor results in a conformation that does not sustain the Glu122-His211 H-bonding interaction due to strong contact between Trp126 and Glu122. Furthermore, the hydrophobic interactions involving Gly121, Leu125, and Phe261 are also maintained in the Meta-II mutant. Consequently, the hydrophobic core of the receptor becomes rigid and distorted, allowing it to make long-range correlated associations with both the N-terminus residues and residues residing in CL3 (Figure S9a). Additionally, the intracellular region of helix 6 is unable to rotate with the mutation-induced interhelical contacts intact. This affects the ability of the active-state intermediate (Meta-II) to be stabilized and it also completely disrupts the signaling pathway that links changes taking place within the ligand-binding pocket during activation (Figure S9a) with helical rearrangements associated with G-protein binding on the IC side of the receptor.

***A269T.*** The mutation of threonine for alanine in position 269 of Meta-II rhodopsin (Ala269Thr or A269T) leads to structural changes on the intracellular side of helix 5 and EL2. Explicitly, the mutation of Ala269 to Thr269 in rhodopsin alters the packing interaction at the helix 6 – helix 7 interface such that the Meta-II stabilizing interaction between Arg135 and Met257 is weakened. In the inactive-state of WT rhodopsin Met257, which resides on the intracellular side of helix 6, is packed against hydrophobic residues Leu128, Leu131, and Ala132 on helix 3 and Asn302 on helix 7. During activation, Met257 moves away from the amphipathic binding pocket defined by Leu128, Leu131 and Asn302 at the IC interface of helices 6 and 7 and new contact interactions are formed between Arg135 and Met257 in Meta-II. The change in receptor interhelical interactions from the perturbation of the mutation at position 269 is reflected in the local structural fluctuations of A269T Meta-II (Figure S9b) when contrasted with the WT Meta-II receptor (Figure 4a). In the LSF of WT Meta-II we find that the Arg135-Met257 interaction is consistent with the outward rotation of H6 and breaking of the dark-state Arg135-Glu247 ionic lock. In the WT receptor, Tyr223 and Tyr306 also form molecular contacts with Met257 and together these additional constraints strengthen the interhelical associations that stabilize the active-state of the receptor. In A269T Meta-II, the destabilizing interactions that are located near the retinal β-ionone ring are transmitted toward the IC side of helix 6 through induced local structural fluctuations. The LSFs in A269T orient the IC region of helix 6 toward the receptor core such that the H-bonding interactions between Met257 and both Tyr223 and Tyr306 are weakened whereas Met257 moves closer to Arg135 (Figure S10c). The overall effect of the A269T mutation is that the IC side of the receptor is held in a less stable active-state conformation. The A269T mutation also has a strong effect on the EC side of the receptor. Due to the large threonine side-chain in the mutant receptor, the β-ionone group of the retinal shifts closer toward helix 5 (Met 207) while the connection between the C20 methyl group of the retinal and Trp265 is maintained in Meta-II (Figure S10d). In WT rhodopsin, the disruption between the C20 methyl group and Trp265 connection in the dark-state is followed by the formation of a new connection between the C19 methyl group and Trp265 in Meta-II. The C19-Trp265 interaction in the active-state receptor is a key step that allosterically couples EL2 and the EC side of helix 5 by forming an essential association between the structural changes taking place on the

extracellular side of the receptor with the formation of the G-protein binding site on the intracellular side. Specifically, the displacement of Trp265 towards the retinal C19 methyl results in an outward motion of the extracellular end of helix 5 that is stabilized by a direct His211-Glu122 H-bonding interaction. The motion of helix 5 positions Tyr223 in a manner such that it able to substitute for Glu247 and disrupt the dark-state ionic-lock (Arg135-Glu247 interaction). Hence, the retention of the C20-Trp265 interaction in A269T Meta-II has ramifications on both the stability of the active-state conformation (Figures S11b and S12b) of rhodopsin and the allosterically coupled motions associated with signal communication (Figure S9b) through the receptor structure. For instance, we find that the failure of the mutant active-state receptor to rupture the C20-Trp265 interaction leads to an uncoupling between helix 5 and EL2 due to the incomplete positioning of Tyr223 in an active conformation. The EC side of helix 5 in A269T Meta-II remains in a destabilized active orientation due to weak coupling between Arg135 and Tyr223 on the IC side of the helix. Moreover, EL2 in the mutant Meta-II receptor also has a distorted configuration due to the weakened constraints on the IC side of the receptor which allows it to make long-range interactions with IC1 (Figure S9b). Previous experimental studies have shown that the Y223F rhodopsin mutant displays a shift in the Meta I - Meta II equilibrium and faster Meta II decay indicating that the changes introduced by this mutation (weaker coupling between Tyr223 and Arg135) are allosterically coupled to the extracellular side of the receptor. From our analysis of the MD simulation of A269T Meta-II, we find that the mutation not only effects the local packing interactions near the retinal binding site but also has long-range communication effects on the activity and functional properties of the entire receptor.

***I189P.*** The Ile189Pro (I189P) mutation in Meta-II rhodopsin is associated with receptor instability that is introduced by a weakened Glu122-His211(H3-H5) interaction. The mutation disrupts the hydrogen-bonding rearrangement in EL2 involving Glu181, Ser186, and Cys187 in Meta-II, which in turn inhibits the rupture of H3/H4/H5 interactions near the retinal pocket that precede the displacement of Trp265 and the helix 6 outward rotation. For instance, an analysis of the MD simulation of I189P reveals that in Meta-II the receptor retains strong interactions between Cys167 (H3) and Met207 (H5) and Thr118 and Glu122 (both in H3), which resemble interhelical interactions found in the crystal structure of the dark-state receptor (Figure S10f). The weaker interaction between Glu122 and His211 in I189P enables the EL region of the retinal binding-site to create long-range interactions with H3, H4, and EL2 that distort the EL structure of the ligand-binding site (Figure S9c). The mutation-induced interhelical interactions impede the movement of Trp265 toward EL2 and subsequently block the rotation of helix 6. The increased number of residue contacts in the EC region of the retinal results in a reduction in the translational dynamics of helices 6 and 7 as well as EL3 (Figures S11c and S12c). The reduction in helical motion facilitates long-range correlations of H6, H7, and EL3 with CL3 and the N-terminus of the receptor (Figure S9c). The mutation of residue 189 in rhodopsin is in EL2 but the influence of the mutation has significant effect on the IC side of the receptor as well. Particularly, we find the mutation is linked with the disruption of a cluster of residues associated with the NPxxY motif. The NPxxY motif is comprised of a collection of residues and internal water molecules that connect the signal propagating dynamics of the retinal with the dynamics taking place on the cytoplasmic

side of the receptor. The helix 6 outward rotation in the active-state receptor moves Met257 away from the hydrophobic pocket of residues on the IC interface of helices 6 and 7 (see description of A269T for more details) in WT Meta-II. The inactive state of the WT receptor also constitutes an interaction amongst residues Asp83 (helix 2), Asn55 (helix 1) and Asn302 (helix 7). The three highly conserved residues (Asn55, Asp83, and Asn302) comprise a H-bonded network in the inactive-state of rhodopsin that is also reorganized in the conversion to Meta-II. The disruption of hydrophobic interactions with Met257 and the conserved residue interactions with Asn302 are replaced with stronger interactions that involve Met257, Asn302 on and Asp83 in the active-state receptor (Figure S10e). The H2/H6/H7 IC interactions stabilize the network of connections that form the NPxxY motif. In 189P Meta-II, the weakened His211-Glu122 interaction on the EC side of the receptor coupled with the hindrance of the helix 6 rotation impedes the association of Met257 with both Asn302 and Asp83 and in effect destabilizes the NPxxY motif and the signal propagation pathway linking the EC and IC side of the receptor (Figure S9c). Also, in I189P the dark-state like linkage between Asn55 and Asp83 is maintained in Meta-II, which distorts the IC side of helix 7 by physically bending it away from the receptor core. Together, the multi-component effects of the single-point mutation of residue189 leads to a likely loss of functional properties of the active-state receptor. Interestingly, the double mutation E122I (Glu122Ile)/I189P (not shown) also modifies both the dynamical and structural properties of Meta-II (when contrasted with the WT receptor) but seemingly maintains the functional properties of the receptor. In this case, the additional substitution of I for E at residue position 22 hampers the ability of Cys167 to strongly couple to residues in helices 3 and 5, which accordingly also permits the H-bonding rearrangement in EL2 to occur - facilitating the helix 6 rotation and the formation of a functional NPxxY pathway. The I122-His211 interaction is considerably weaker than the WT Glu122 -His211 association, hence the overall thermal stability of the double-mutant receptor is considerably altered than the WT receptor.

***F261Y***. The Phe261Tyr (F261Y) mutation in Meta-II alters the hydrophobic interactions in the IC region of the active-state receptor. Residues such as Gly121, Leu125, Leu128, Leu131, and Ala132 on helix 3 and Phe261 and Trp265 on helix 6 that form a micro-domain in the interior of rhodopsin are altered with the single point mutation. The residue interactions forming the hydrophobic domain are modified during the transformation from the inactive- to active-state intermediate (Meta-II). In fact, the modifications of these hydrophobic interactions on the IC side of the receptor are intimately tied with transmitting the conformational changes taking place in the retinal-binding pocket with intra-protein interactions taking place on the cytoplasmic side of the receptor during activation. In the F261Y receptor, the mutation alters the active-state interactions of the cluster of hydrophobic residues such that the shape of the retinal-binding pocket is considerably deformed. Specifically, in F261Y we find that contact interactions between Gly121, Leu125, and Phe261 are enhanced in the Meta-II state (when contrasted with WT Meta-II), whereas the mutation maintains a weaker interaction between His211 and Glu122 (Figure S10g). The increased interaction between the hydrophobic residues in the IC region of the receptor are evident in the LSF of F261Y in Figure S9d. We also notice in the LSF of F261Y that there is a stronger coupling between residues in helices 6, 7, and EL3 that form the signal activation pathway in

Meta-II and the N-terminus of the receptor when compared with the WT receptor (Figure 4a). The deformation of the ligand-binding site mediates stronger interactions between residues in helix 4 and EL2, which in turn shifts the EC side of the retinal pocket closer to the N-terminus. The shift in the position of the retinal pocket creates a larger separation between the retinal and EL2. It is interesting to note that helix 4 residues are coupled with rearrangements that are necessary for rhodopsin activation. For instance, Cys167 in helix 4 (which resides in close proximity to the β-ionone ring) is tightly coupled to Met207 (helix5) and Thr118 (helix3) in the inactive-state. During isomerization, the interaction with these residues must be displaced to make space for isomerized β-ionone ring. The change in the H3/H4/H5 interactions that accompany the isomerized retinal are also associated with intramolecular associations that induce helical rearrangements that enable helix 5 to adopt an active conformation in Meta-II. The active-state changes in interhelical interactions also maintain the active-state of the receptor. In F261Y, the EL2 - helix 4 correlated motion (Figure S9d) destabilizes the activation pathway that connects the dynamics taking place within the retinal pocket with the dynamics occurring in the cytoplasmic side of the receptor. This, coupled with the decreased retinal-protein interactions in the region of Glu122 (H3) and His211 (H5) in F261Y contribute to the overall weaker coupling between allosteric components associated with the activation mechanism in the receptor.

*Double mutations and receptor thermal stability in Meta-II.*

***A269T/I189P***. In an analysis of the MD simulation of A269T/I189P we find that the double mutation introduces new correlations that connect the dynamics in the ligand-binding site with the motion of residues in EL2 and EL3. These new correlations are present in neither the WT Meta-II receptor or in the single mutant receptors A269T or I189P. Specifically, the I189P mutation in EL2 creates packing defects around the ligand-binding site. In the previous discussion regarding the single point mutation (I189P), we pointed out that the ECL mutation forms contact associations near the b-ionone ring that result in a weakened H-bonding interaction between His211 and Glu122. In the double mutant receptor, the addition of the A269T mutation counters the ECL-induced packing defects by introducing altered retinal-protein interactions that are also induced by the I189P ECL mutation. In the single-mutant A269T receptor, contact between the C20 retinal methyl group and Trp265 obstructed the helix 6 rotation. As was mentioned in previous sections, the helix 6 rotation is required for the receptor to assume an active conformation. The I189P mutation modifies the retinal-Trp265 contact associations in the double mutant receptor such that the ability of the receptor to support the helix 6 outward rotation is restored. Accordingly, the interaction between Glu122 and His211 is reinforced on the EC side of the retinal. The restored H6 rotation also moves Met257 closer to Asn302 on the IC side of the retinal, reestablishing the disrupted interactions in the NPxxY motif that obstructed signal communication in both single-point mutants (I189P and A269T). The helix 6 motion coupled with the rearrangement in residue interactions in the ligand-binding site create long-range interactions among H3, H4, H6, H7, EL2 and EL3 (Figure S9e). The long-range associations shift the EC side of ligand-binding pocket away from the retinal, creating a less protected ligand-binding site and the likely increase in the rate of hydrolysis of the PSB when contrasted with WT rhodopsin.

The more exposed retinal-binding pocket also creates a weaker allosteric connection from the EC to the IC side of the receptor.

***M163W/I189P***. As with the A269T/I189P double mutation, the receptor EC modified interactions stemming from the I189P mutation result in a series of packing defect interactions near the b-ionone ring that decrease receptor stability on the EC side of the ligand-binding site. On the other hand, the M163W mutation generates packing irregularities in the H3/H6 association that ultimately effects the ability of the receptor to adopt an active conformation. The construction of a double mutant Meta-II receptor containing both A269T and I189P reshapes the ligand-binding site by facilitating helix-helix interactions at the H3-H5 interface (Figure S9f), which reflect increased retinal-protein interactions in the region of Glu122 (H3) and His211(H5). However, the aromatic cluster of amino acids at the extracellular end of H6 involving Phe261, Trp265, and Tyr268 is retained in M163W/I189P Meta-II. This prevents the motion of Trp265 and consequently the motion of H6 and the ability of the receptor to adopt a stable, Meta-II conformation. The retention of the aromatic cluster in the Meta-II double-mutant receptor creates a rigid EC end of helix 6 that supports long-range correlated associations with residues in EL1, EL2, EL3, and CL3 (Figure S9f, S11f, and S12f). The long-range interactions of the EC side of the receptor with residues in the intracellular loop region distorts the ligand-binding site such that the allosterically coupled motions connecting the EC side of the receptor with the IC side is inhibited, leading to the possible loss of function of receptor activity.

***F261Y/A269T***. In the F261Y/A269T Meta-II MD simulation, we find increased interaction between Thr269 and Trp265 in the double mutant receptor that disrupts the hydrophobic cluster of aromatic residues created due to the F261Y mutation. The intra-helical interaction between Thr269 and Trp265 reorients helix 6 such that the IC side of the helix creates stronger interactions between helices 6 and 7 (Met257 – Asn302), which in turn reinforces the NPxxY motif connecting the ligand-binding dynamics with residue interactions in the cytoplasmic region. The induced interactions near the b-ionone ring also shifts the EC side of the ligand-binding site closer to EL2 by promoting stronger retinal interactions with Thr118 in helix 3, Tyr268 in helix 6, and Ile189 on EL2. Interestingly, Ile189 points directly in the direction of Pro291 and with the aid of conserved water molecules connects EL2 with the EC region of helix 7. Altogether, the double mutant active-state receptor promotes interhelical interactions in the EC region of the receptor that couple the dynamics in EL1, EL2, EL3, H6, and H7 (Figure S9h). The EC interhelical associations create an elongated retinal-binding site with a larger separation between the retinal and EL2 region when compared with WT rhodopsin. Further, the induced retinal-protein interactions facilitate enhanced, long-range signaling in the network of connected residues (Figure 8) that are valuable in translating augmented retinal conformational changes that take place during activation in the ligand-binding site into helical rearrangements in the intracellular region of the receptor that are propagated to the cytoplasmic surface.

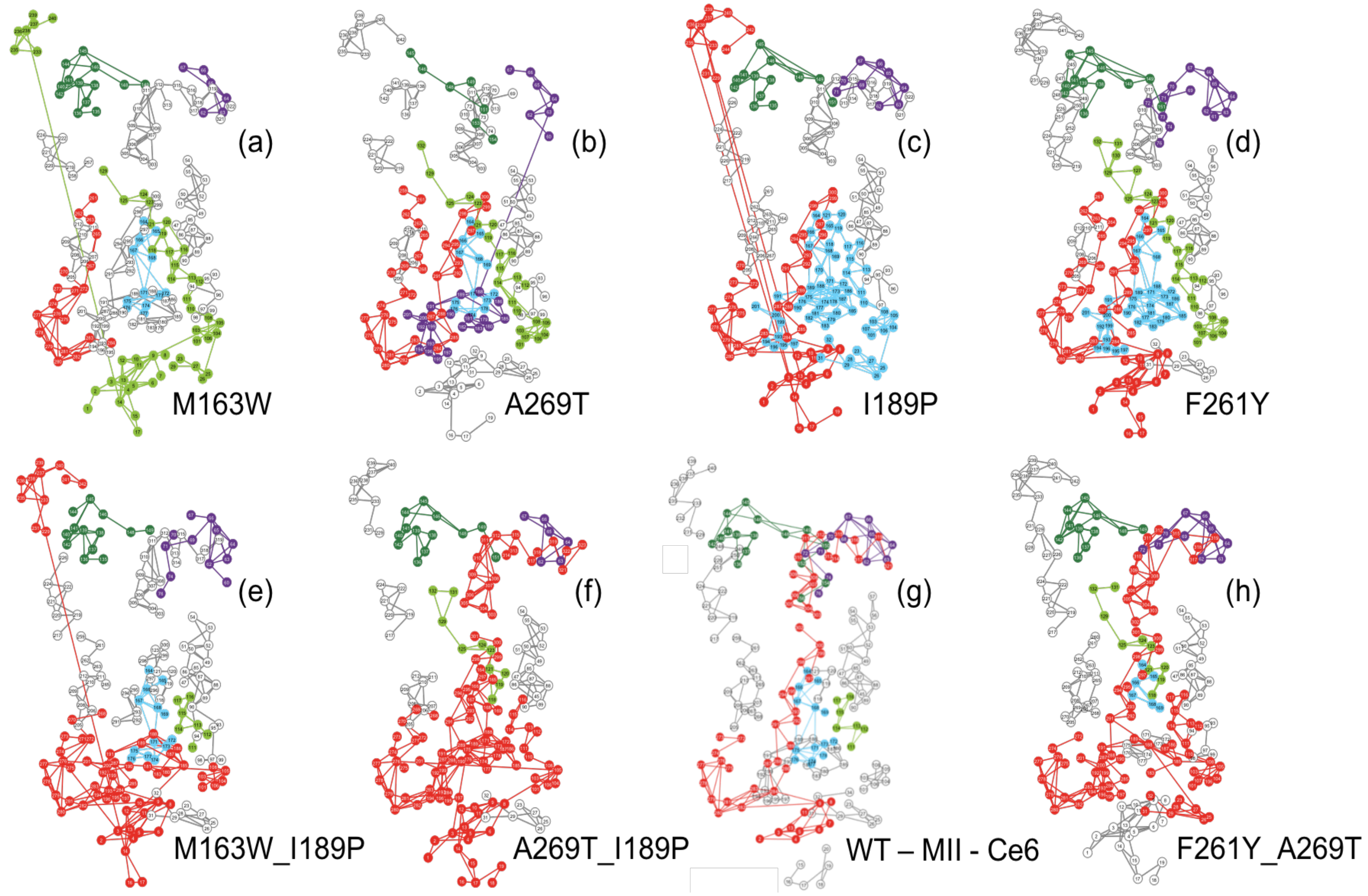


**Figure S9:** A 2-D network mapping of the LSFs from the MD simulation of (**a**) M163W Meta-II, (**b**) A269T Meta-II, (**c**) I189P Meta-II, (**d**) F261Y Meta-II, (**e**) M163W_I189P Meta-II, (**f**) A269T_I189P Meta-II, (**g**) WT Meta-II bound to Ce6, and (**h**) F261Y_A269T Meta-II.

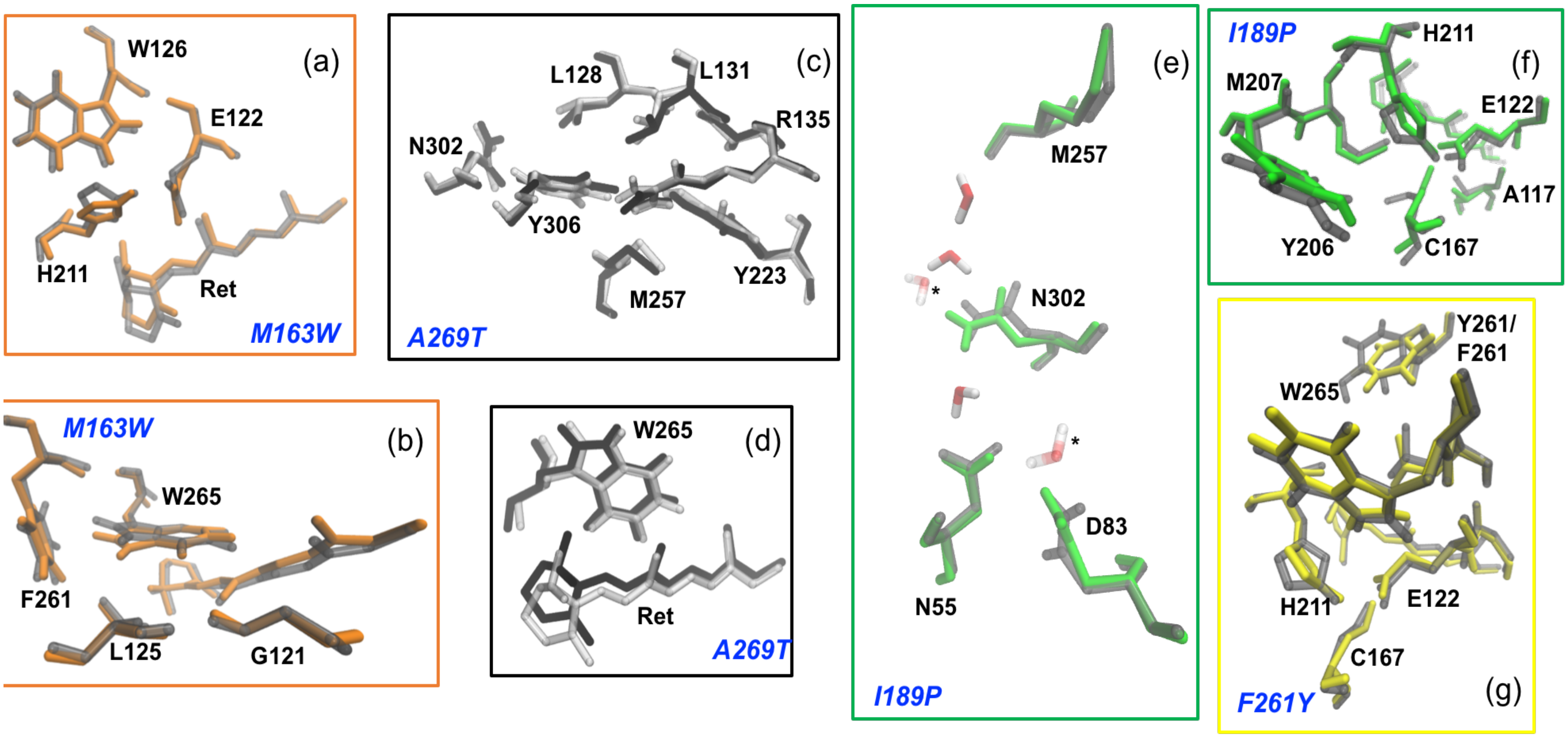


**Figure S10**: Snapshots of residue and retinal-residue interactions from the MD simulations of mutants of Meta-II rhodopsin contrasted with residue interactions from WT Meta-II. In all cases, the residues from the WT simulation are colored whereas the residues from the mutant receptors are colored in transparent gray (or transparent in general in the case of mutant receptor water molecules which are also labeled with an asterisk).

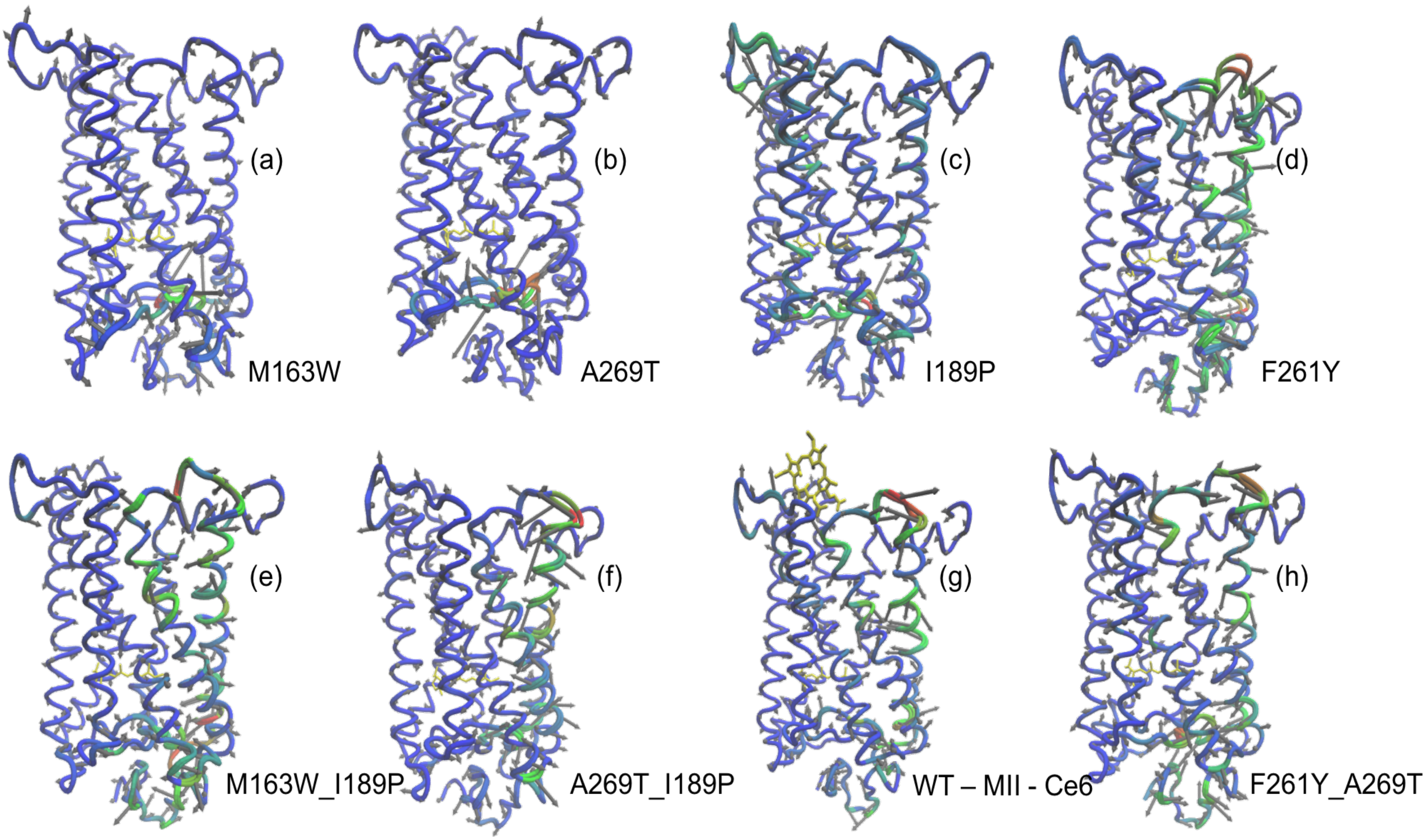


**Figure S11**: C-α representation of the dominant PCA mode (PCA1) from the MD simulation of (**a**) M163W Meta-II, (**b**) A269T Meta-II, (**c**) I189P Meta-II, (**d**) F261Y Meta-II, (**e**) M163W_I189P Meta-II, (**f**) A269T_I189P Meta-II, (**g**) WT Meta-II bound to Ce6, and (**h**) F261Y_A269T Meta-II. Areas colored in red are associated with more mobile regions in the receptor structure, whereas areas colored in blue correspond to more rigid regions.

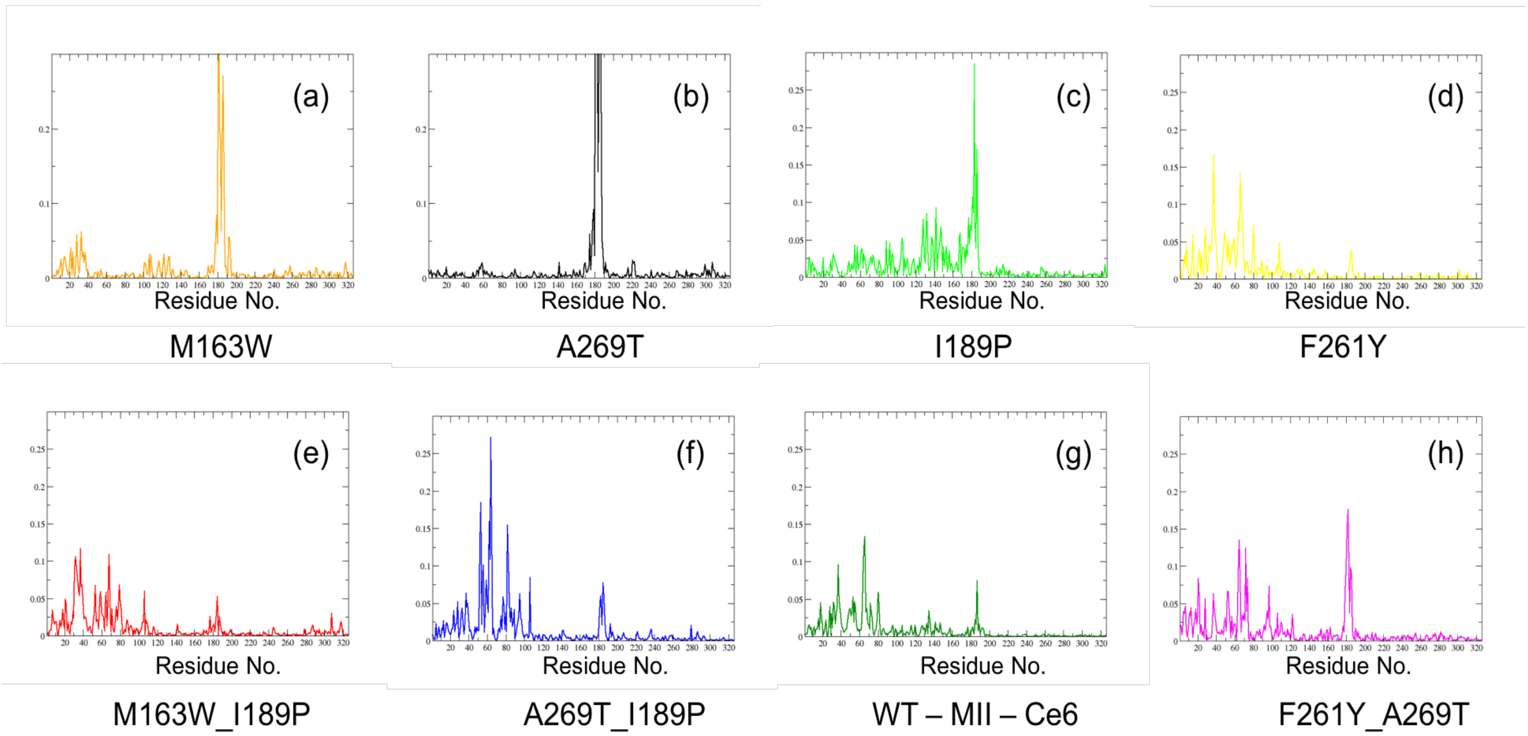


**Figure S12**: The per residue RMSD from the MD simulation analysis of the dominant PCA mode (PCA1) of (**a**) M163W Meta-II, (**b**) A269T Meta-II, (**c**) I189P Meta-II, (**d**) F261Y Meta-II, (**e**) M163W_I189P Meta-II, (**f**) A269T_I189P Meta-II, (**g**) WT Meta-II bound to Ce6, and (h) F261Y_A269T Meta-II.

**Supplementary text, *Ce6 modulation of GPCR-A conserved allosteric sites in rhodopsin***

*Ce6 modulation of conserved allosteric interactions coupling the ligand-binding site with the ECD in rhodopsin-like receptors*

From our MD simulations, we have deduced that Ce6 binding in active-state rhodopsin alters the allosteric coupling between the receptor ligand-binding site and the ECD. To shed further light on this phenomenon and to predict how Ce6 may act on other GPCR-A receptors, a network representation (Figure 8) of a multiple sequence alignment (MSA) of the rhodopsin-like family of receptors (GPCR-A) has been constructed. The network reveals that Ala269$^{6.52}$ at the EC side of helix 6 is a residue with relatively high coevolution propensity that forms a link with another coevolved residue, Ile189$^{45.52}$ in EL2. Previously, we have indicated that both residues have a prominent role in altering the activation pathway in Meta-II. A closer inspection of the network representation of the MSA reveals that the coevolved residue pairs (Ala269$^{6.52}$ and Ile189$^{45.52}$) are in separate network communities (Figure 8 and Supplementary Figure S14). In general, coevolved residues that serve as "linkers" between different network communities like Ala269$^{6.52}$ have been shown to facilitate enhanced, long-range signaling in the network of connected residues. In this particular case, the Ala269$^{6.52}$ – Ile189$^{45.52}$ connection represents a conserved link between the ECD and ligand-binding site in GPCR-A

receptors. The pathway of their connection signifies a correlation between the dynamics in the ligand-binding site and the motion of residues in EL2 and EL3 in the ECD. Rhodopsin is known for having a low thermal activation rate (low dark-noise), which permits single-photon photosensitivity. This is attributed to the position of the receptor's unique EL2 positioning (5,6). The EL2 in rhodopsin is positioned at the opening of the ligand-binding pocket and serves as a stable "lid" to block rapid exit of the ligand from the pocket. However other known class-A receptors use multiple ligands and hence, possess a more structurally "open" binding-pocket that facilitates ligand exchange. For example, both the $\beta_1$- adrenergic (ADRB1) and $\beta_2$- adrenergic (ADRB2) receptors have a greater number of polar and aliphatic residues in EL2 and EL3 when contrasted with rhodopsin. Adrenergic receptors are also known to have much higher basal activity. The difference is accredited to the fact that both adrenergic receptors bind a diverse number of diffusible ligands (7–11) that must make their way in and out of the ligand-binding pocket of the receptor via the extracellular region, whereas rhodopsin covalently binds retinal in its ligand-binding pocket. Hence, there is a greater amount of conformational mobility in the extracellular region of the active- and intermediate- states of ADRB1 and ADRB2 when compared with the active-like states of rhodopsin (12). MD simulations of a cone-pigment has revealed that a substitution of a polar amino acid with a bulky side-chain at position 269 (when contrasted with bovine rhodopsin) has an overall disruptive effect on the structural stability of the EC side of the ligand-binding site. Interestingly, we have observed an analogous effect on the general structural flexibility of the EC region of Meta-II rhodopsin when bound with Ce6. In our MD simulations, we have noted that Ce6 binds explicitly in CL2 (residues 140 – 150 in rhodopsin) in Meta-II. Essentially, Ce6-binding in rhodopsin appears to act on a GPCR-A conserved allosteric site that alters the coupling mechanism between the ECD and ligand-binding site (Figure 8). In rhodopsin, we propose that this action has a strong effect on the thermal stability of the entire receptor and may act on other GPCR-A receptors in a similar manner.

*Ce6-induced modifications of conserved allosteric sites coupling the ligand-binding site and the IC region of GPCR-A receptors*

Ce6 binding in rhodopsin also influences GPCR-A allosteric sites that regulate coupling between the IC region and the retinal ligand-binding site. Through binding and residue contacts in CL2, Ce6 modulates GPCR-A allosteric interactions that adjust the pathway from the retinal-binding pocket to the IC side of the receptor. This is more explicitly depicted in the network representation of the MSA of the rhodopsin-like family of GPCRs in Supplementary Figure S14 and Figure 8. Specifically, we show that in the network mapping of class-A GPCR interactions (Supplementary Figure S14) that Pro267$^{6.50}$ serves as a "linker" residue between the ligand-binding site and Asn302$^{7.49}$, which forms part of the conserved NPxxY motif found in the intracellular region of the receptor. An illustrated network of these interactions in the MSA in Figure 8 describes a series of conformational changes in the GPCR-A family of receptors that occur in response to activation: (*i*) hydrophobic contact changes are initiated in the ligand-binding pocket (Phe261$^{6.44}$) after activation that are (*ii*) subsequently transmitted to the IC region by the rigid body rotation of helix 6 at the cytoplasmic end of Pro267$^{6.50}$. From the IC region, the signal is further propagated through a (*iii*) conserved network of H-bonding interactions (Asn302$^{7.49}$) toward the cytoplasmic side where G-protein binding occurs.

These steps represent common elements of activation in the GPCR-A class of receptors. We observe that Ce6 binding in CL2 of rhodopsin explicitly alters the dynamic correlations and coevolutionary residue couplings that direct the organization of allosteric interactions that mediate this shared mechanism of activation in the family of rhodopsin-like receptors. For instance, we observe in our MD simulations of Meta-II that Ce6-binding specifically modifies the contact interactions between Gly121$^{3.36}$, Leu125$^{3.40}$, and Phe261$^{6.44}$. This cluster of residues forms a hydrophobic domain in the 7TM interior that is a shared feature in all known class-A GPCRs. These hydrophobic associations govern the packing interactions between helices 3 and 6 on the IC side of the receptor, and changes in these interhelical associations are a trigger for the motion of helix 6 during activation. Induced fluctuations stemming from the binding of Ce6 in Meta-II lead to the loss of a conserved water molecules (Wat1) on the IC side of the receptor that particularly diminishes the translational mobility of Phe261$^{6.44}$ and consequently hinders the complete disruption of the hydrophobic cluster of residues during activation. Phe261$^{6.44}$ is a moderately conserved residue in the MSA but possess relatively high coevolutionary propensity (MI value). This coupled with its connection to other high MI value residues suggests that Phe261$^{6.44}$ has a critical functional role in the signal propagating mechanism in the family of receptors. Our findings indicate that Ce6 particularly alters the conserved architecture of rhodopsin-like GPCR hydrogen-bonding contacts that regulate the translation of conformational changes taking place at the ligand-binding site with those taking place at the receptor cytoplasmic surface. Accordingly, the moderated contacts have a direct effect on both ligand-binding and G-protein binding affinity.

*Ce6-induced alterations in conserved allosteric associations linking the ECD, ligand-binding site, and G-protein binding site in GPCR-A receptors*

The relationship between the Ce6-induced interactions in the IC region of rhodopsin and the significance of Met163$^{4.52}$ in rhodopsin (as well as other class-A receptors) in establishing critical constraints that maintain the active-state conformation of the receptor can also be deduced by evaluating the network of interactions from the MSA of the GPCR-A family of receptors in Figure 8. From the alignment, it becomes apparent that Met163$^{4.52}$ has prominent connections to coevolved residue pairs forming the ERY motif (Glu134$^{3.49}$-Arg135$^{3.50}$-Tyr136$^{3.51}$) that reside near the cytoplasmic side of TM3. The conserved residue interactions are associated with regulating the helix 3 - helix 6 distance after activation and in turn, supporting the active-state signaling pathway that determines both G-protein activity and signal amplification. The GPCR-A alignment also provides further insight into the role of Met163$^{4.52}$ in establishing long-range allosteric connections in the active-state protein that regulate the ability of the receptor to accommodate the G-protein. In the sequence alignment, one can clearly discern the network of interactions within the same community (as Met163$^{4.52}$) that form links to residues in CL2 and CL3. The connected residues in CL2 and CL3 have an established role in creating the protein-protein binding interface at the surface of the cytoplasmic domain. The dense network of contacts involving Met163$^{4.52}$ (Figure 8) reflects the allosterically coupled set of interactions between the ligand-binding site and the G-protein binding site in the class-A family of receptors.

Figure 8 also highlights the close association of Met163$^{4.52}$ with Ile189$^{45.52}$ in the network representation of the GPCR-A MSA. In our investigation on the correlated fluctuations in Meta-II from the MD simulations (Figure 4) we determined that Met163$^{4.52}$ has a prominent role in governing the distance between EL2 and ligand-binding site. In Meta-II-Ce6 this distance is extended due to the structural changes that take place with Ce6-binding in the intracellular region of the receptor. Based on the results of our MD simulations on Meta-II-Ce6, we also observe that Ce6 binding in CL2 of the active-state receptor directly modulates the interhelical H-bonds surrounding Met163$^{4.52}$ (Supplementary text, *MD simulation of Meta-II mutations*, Supplementary Figures S9a) that determine the conformational dynamics of TM5. One of the more distinct changes that take place in class-A receptors upon activation is the elongation of the cytoplasmic end of helix 5 in the formation of the G-protein binding site at the CP interface. Stabilization and binding-affinity of the G-protein binding interface is moderated by (*i*) residues that form H-bonds with the agonist at the EC side of the ligand-binding site (Glu122$^{3.37}$ and His211$^{5.46}$) and (*ii*) hydrophobic packing changes (involving Leu125$^{3.40}$, Pro215$^{5.50}$, and Phe261$^{6.44}$) on the intracellular side of the ligand-binding site that shifts the IC end of helix 6 away from helix 3. The latter maintains the rupture of the ionic-lock formed by the D(E)RY motif. Upon activation, Met163$^{4.52}$ interaction with Glu122$^{3.37}$ stabilizes the bend initiated by interhelical changes involving Pro215$^{5.50}$ on helix 5. The active-state H3/H4/H5 interaction intersected by Met163$^{4.52}$ is simultaneously correlated with hydrogen-bonding rearrangements in EL2 involving Ile189$^{45.52}$ in the active-state receptor. It is interesting to point out that residue 4.52 (Met163 in rhodopsin) is moderately conserved in the alignment of class-A receptors (42%), and in all receptors comprising the MSA, Met163$^{4.52}$ is a hydrophobic residue with an aliphatic side-chain. This, together with the relatively high MI value of Met163$^{4.52}$ suggests that the residue holds a strategic structural position amongst the coevolved residue pairs that create a signaling pathway through the class-A receptors. We find that Met163$^{4.52}$ in rhodopsin plays a pivotal role in *correlating* the associations that form the distinct allosteric network of interactions that link the EC side of the ligand-binding site with those in the IC region in the formation of the G-protein binding surface. Specifically, it links changes taking place near the ligand during activation with a complex arrangement of long-range interactions to residues in the intracellular region that rearrange to form the G-protein binding-site. Ce6 binding weakens these GPCR-A family-wide conserved connections by decoupling the individual components of the allosteric network. The destabilized network of interactions coupling the individual components of the allosteric cluster of residues promotes a structurally heterogeneous active-state with a decreased capacity to bind the G-protein. Our observations are in line with previous structural studies on rhodopsin that have identified interhelical interactions involving Met163$^{452}$ (H4): Glu122$^{3.37}$ (H3): His211$^{5.46}$ (H5) that provide essential contacts that retain core interactions in the active-state receptor (13–15).

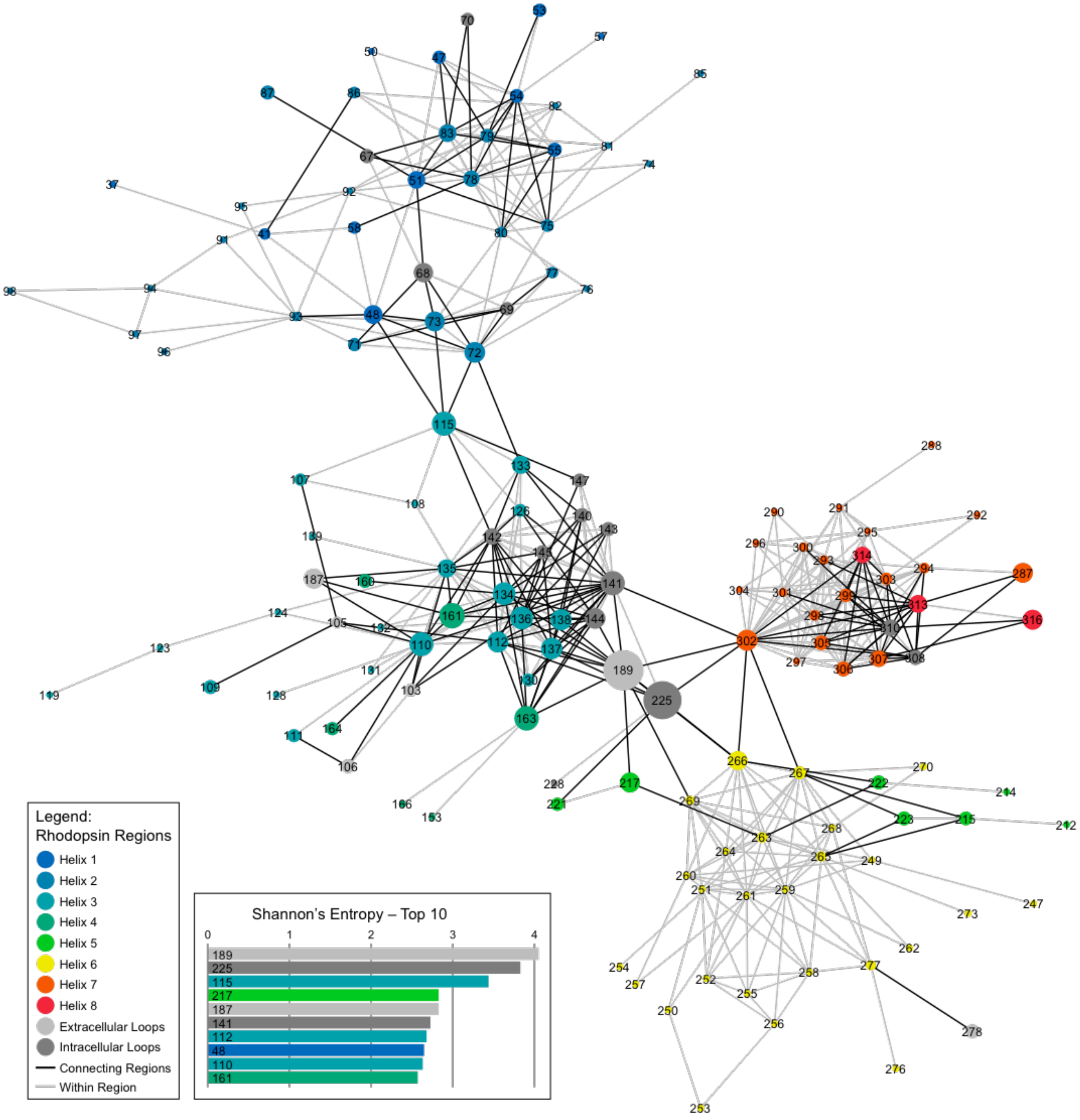


**Figure S13**: Network representation of the MSA of rhodopsin-like (class -A) family of proteins where in this case the colors represent the different secondary structural regions of rhodopsin and the size of nodes depict Shannon's Entropy of the node with respect to its connected neighbors in the MSA network. The nodes with the largest values are shown in the bottom chart and illuminate the importance of these particular residues in the evolution of the receptor family.

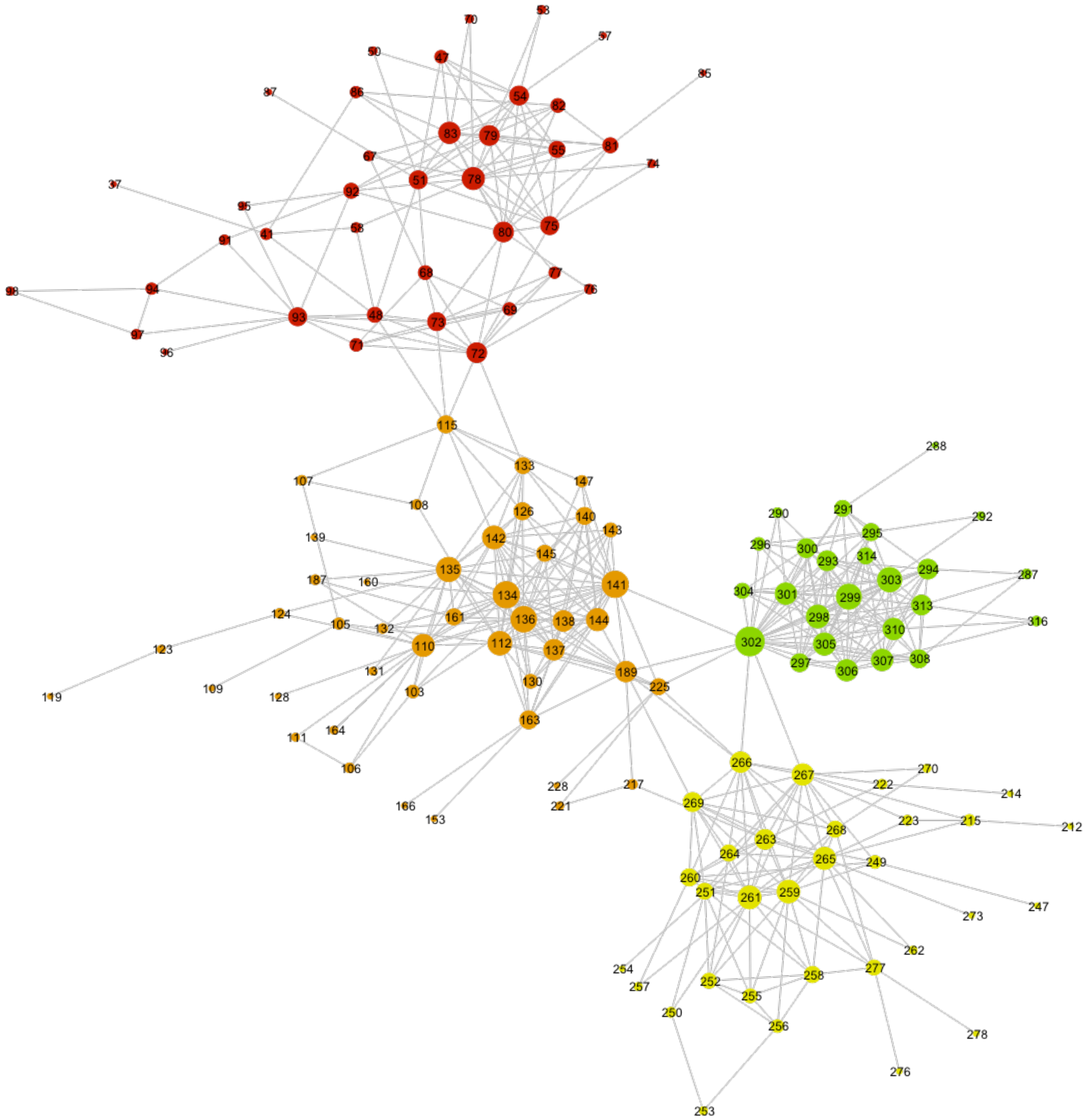


**Figure S14**: MSA of the rhodopsin-like family of proteins broken into communities with each community containing different colors. In communities, nodes within the same community are highly connected and communicate with one another efficiently in a localized manner, whereas nodes in different communities have fewer connections and require *intermodular edges* (or intermoldular links) for forming long-distance communication. The nodes represent amino acid residues, the links are the connections between the residues, and the size of the nodes denotes the number of connections (links) to other nodes. The reference structure for the MSA is bovine rhodopsin (opsd_bovin) with pdb ID 1u19.

**References**


1. Werner K, Richter C, Klein-Seetharaman J, Schwalbe H. Isotope labeling of mammalian GPCRs in HEK293 cells and characterization of the C-terminus of bovine rhodopsin by high resolution liquid NMR spectroscopy. *J Biomol NMR* (2008) **40**:49–53. doi:10.1007/s10858-007-9205-3

2. Boyce SE, Mobley DL, Rocklin G, Graves AP, Dill KA, Shoichet BK. Predicting ligand binding affinity with alchemical free energy methods in a polar model binding site. *J Mol Biol* (2009) **394**:747–763. doi:10.1016/j.jmb.2009.09.049

3. Hornak V, Ahuja S, Eilers M, Goncalves JA, Sheves M, Reeves PJ, Smith SO. Light activation of rhodopsin: insights from molecular dynamics simulations guided by solid-state NMR distance restraints. *J Mol Biol* (2010) **396**:510. doi:10.1016/j.jmb.2009.12.003

4. Kirchberg K, Kim T-Y, Möller M, Skegro D, Dasara Raju G, Granzin J, Büldt G, Schlesinger R, Alexiev U. Conformational dynamics of helix 8 in the GPCR rhodopsin controls arrestin activation in the desensitization process. *Proc Natl Acad Sci U S A* (2011) **108**:18690–18695. doi:10.1073/pnas.1015461108

5. Janz JM, Fay JF, Farrens DL. Stability of Dark State Rhodopsin Is Mediated by a Conserved Ion Pair in Intradiscal Loop E-2. *J Biol Chem* (2003) **278**:16982–16991. doi:10.1074/jbc.M210567200

6. Yanagawa M, Kojima K, Yamashita T, Imamoto Y, Matsuyama T, Nakanishi K, Yamano Y, Wada A, Sako Y, Shichida Y. Origin of the low thermal isomerization rate of rhodopsin chromophore. *Sci Rep* (2015) **5**:11081. doi:10.1038/srep11081

7. Dror RO, Arlow DH, Borhani DW, Jensen MØ, Piana S, Shaw DE. Identification of two distinct inactive conformations of the β2-adrenergic receptor reconciles structural and biochemical observations. *Proc Natl Acad Sci* (2009) **106**:4689–4694. doi:10.1073/pnas.0811065106

8. Lamichhane R, Liu JJ, Pljevaljcic G, White KL, van der Schans E, Katritch V, Stevens RC, Wüthrich K, Millar DP. Single-molecule view of basal activity and activation mechanisms of the G protein-coupled receptor β2AR. *Proc Natl Acad Sci U S A* (2015) **112**:14254–14259. doi:10.1073/pnas.1519626112

9. Manglik A, Kim TH, Masureel M, Altenbach C, Yang Z, Hilger D, Lerch MT, Kobilka TS, Thian FS, Hubbell WL, et al. Structural Insights into the Dynamic Process of β2-Adrenergic Receptor Signaling. *Cell* (2015) **161**:1101–1111. doi:10.1016/j.cell.2015.04.043

10. Manglik A, Kobilka B. The role of protein dynamics in GPCR function: insights from the β2AR and rhodopsin. *Curr Opin Cell Biol* (2014) **0**:136–143. doi:10.1016/j.ceb.2014.01.008

11. Kahsai AW, Xiao K, Rajagopal S, Ahn S, Shukla AK, Sun J, Oas TG, Lefkowitz RJ. Multiple ligand-specific conformations of the β2-adrenergic receptor. *Nat Chem Biol* (2011) **7**:692–700. doi:10.1038/nchembio.634

12. Dror RO, Arlow DH, Maragakis P, Mildorf TJ, Pan AC, Xu H, Borhani DW, Shaw DE. Activation mechanism of the β2-adrenergic receptor. *Proc Natl Acad Sci* (2011) **108**:18684–18689. doi:10.1073/pnas.1110499108

13. Ahuja S, Crocker E, Eilers M, Hornak V, Hirshfeld A, Ziliox M, Syrett N, Reeves PJ, Khorana HG, Sheves M, et al. Location of the Retinal Chromophore in the Activated State of Rhodopsin(*). *J Biol Chem* (2009) **284**:10190–10201. doi:10.1074/jbc.M805725200

14. Menon ST, Han M, Sakmar TP. Rhodopsin: structural basis of molecular physiology. *Physiol Rev* (2001) **81**:1659–1688.

15. Palczewski K, Kumasaka T, Hori T, Behnke CA, Motoshima H, Fox BA, Le Trong I, Teller DC, Okada T, Stenkamp RE, et al. Crystal structure of rhodopsin: A G protein-coupled receptor. *Science* (2000) **289**:739–745.
